# Synergistic strategy with hyperthermia therapy based immunotherapy and engineered exosomes−liposomes targeted chemotherapy prevents tumor recurrence and metastasis in advanced breast cancer

**DOI:** 10.1002/btm2.10284

**Published:** 2021-12-27

**Authors:** Haiqin Huang, Lanlan Shao, Yan Chen, Lan Tang, Tianqing Liu, Junxu Li, Hongyan Zhu

**Affiliations:** ^1^ Department of Pharmaceutics School of Pharmacy, Nantong University Nantong China; ^2^ NICM Health Research Institute Western Sydney University Westmead New South Wales Australia

**Keywords:** antitumor immune response, GNR, TEX‐liposome‐PTX, thermal ablation

## Abstract

Advanced breast cancer with recurrent and distal organ metastasis is aggressive and incurable. The current existing treatment strategies for advanced breast cancer are difficult to achieve synergistic treatment of recurrent tumors and distant metastasis, resulting in poor clinical outcomes. Herein, a synergistic therapy strategy composed of biomimetic tumor‐derived exosomes (TEX)‐Liposome‐paclitaxel (PTX) with lung homing properties and gold nanorods (GNR)‐PEG, was designed, respectively. GNR‐PEG, with well biocompatibility, cured recurrent tumors effectively by thermal ablation under the in situ NIR irradiation. Meanwhile, GNR‐mediated thermal ablation activated the adaptive antitumor immune response, significantly increased the level of CD8^+^ T cells in lungs and the concentration of serum cytokines (tumor necrosis factor‐α, interlekin‐6, and interferon‐γ). Subsequently, TEX‐Liposome‐PTX preferentially accumulated in lung tissues due to autologous tumor‐derived TEX with inherent specific affinity to lung, resulting in a better therapeutic effect on lung metastasis tumors with the assistance of adaptive immunotherapy triggered by GNR in vivo. The enhanced therapeutic efficacy in advanced breast cancer was a combination of thermal ablation, adaptive antitumor immunotherapy, and targeted PTX chemotherapy. Hence, the synergistic strategy based on GNR and TEX‐Liposome provides selectivity to clinical treatment of advanced breast cancer with recurrent and metastasis.

## 1 INTRODUCTION

As reported, advanced breast cancer possesses the characteristics of refractory, malignant invasion^1^ and poor prognosis.^2^, ^3^ Patients with advanced breast cancer have a shorter survival time, and the mortality rate is approximately 90% within 5 years after diagnosis.^4^, ^5^, ^6^ The recurrent tumors or distant organs metastasis (lung, bone) can be produced via system blood, forming advanced breast cancer. Current clinical oncologic methodolodies for advanced breast cancer mainly include local treatment, immunotherapy and systemic chemotherapy, including single or combination chemotherapy.^7^ Generally, existing treatments do not achieve complete cure and usually end in failure due to the insurmountable targeting issues, drug resistance and systemic side effects. Meanwhile, patients in the advanced stage are poorly tolerated, leading to further failure in clinical. Thus, an innovative and effective precise treatment strategy for advanced breast cancer with recurrent tumors and distal organ metastasis is urgently needed, aiming to achieve maximum efficacy accompanied with minimum toxicity.

Nanomaterial‐based photothermal therapy (PTT) has been extensively explored as an emerging strategy against malignant tumors in clinical.^8^ Compared with conventional treatment such as surgical resection, radiotherapy and chemotherapy, patients with advanced tumors have higher compliance with PTT. As reported, various nanomaterials, such as gold nanorods (GNR),^9^, ^10^, ^11^, ^12^ graphene oxide,^13^ carbon nanotubes,^14^ and indocyanine green, have been widely explored for PTT. As highly effective and noninvasive treatment models, PTT has a direct cancer cell killing effect for local tumors through hyperthermia ablation.^15^, ^16^, ^17^, ^18^, ^19^ Simultaneously, hyperthermia ablation of tumors can release tumor‐associated antigens and these antigens are recognized by dendritic cells (DC) and presented to native T cells, thus promoting the activation of adaptive antitumor immune responses, and further enhancing antitumor efficacy through immunetherapy.^20^, ^21^ Although many investigations have demonstrated the remarkable antitumor efficacy of PTT combined with immunotherapy,^22^, ^23^ it is not able to cure the distal metastasis lesions.^24^

Currently, most of the distant metastatic tumors are treated by systemic chemotherapy in clinical. However, lack of active targeting property makes the treatment need to face the situation of drug resistance, side effects, and failure. Exosomes, with a bilayer membrane‐like structure similar to liposome,^25^, ^26^, ^27^ are naturally secreted by most cell types.^28^ As reported, exosomes secreted by breast cancer cells possess excellent lung targeting capability due to their functional surface integrins (α6β4, α6β1),^29^ which colocate in the laminin‐rich lung microenvironment.^30^, ^31^ The characteristic endows enormous potential for employing exosomes as targeted drug delivery vehicles.^32^, ^33^ In the early stage, exosomes derived from breast cancer were adopted as a gene delivery vector for the delivery of siS100A4, exhibiting excellent lung targeting capability and realizing the treatment of lung metastasis.^34^ However, the low drug loading efficiency of exosomes limits their potential applications as drug delivery platforms.^35^ To overcome the targeting defects of lipid‐based vectors, a biomimetic hybrid system composed with tumor‐derived exosomes (TEX) and liposomes for specific delivery to lung was proposed.

**SCHEME 1:**
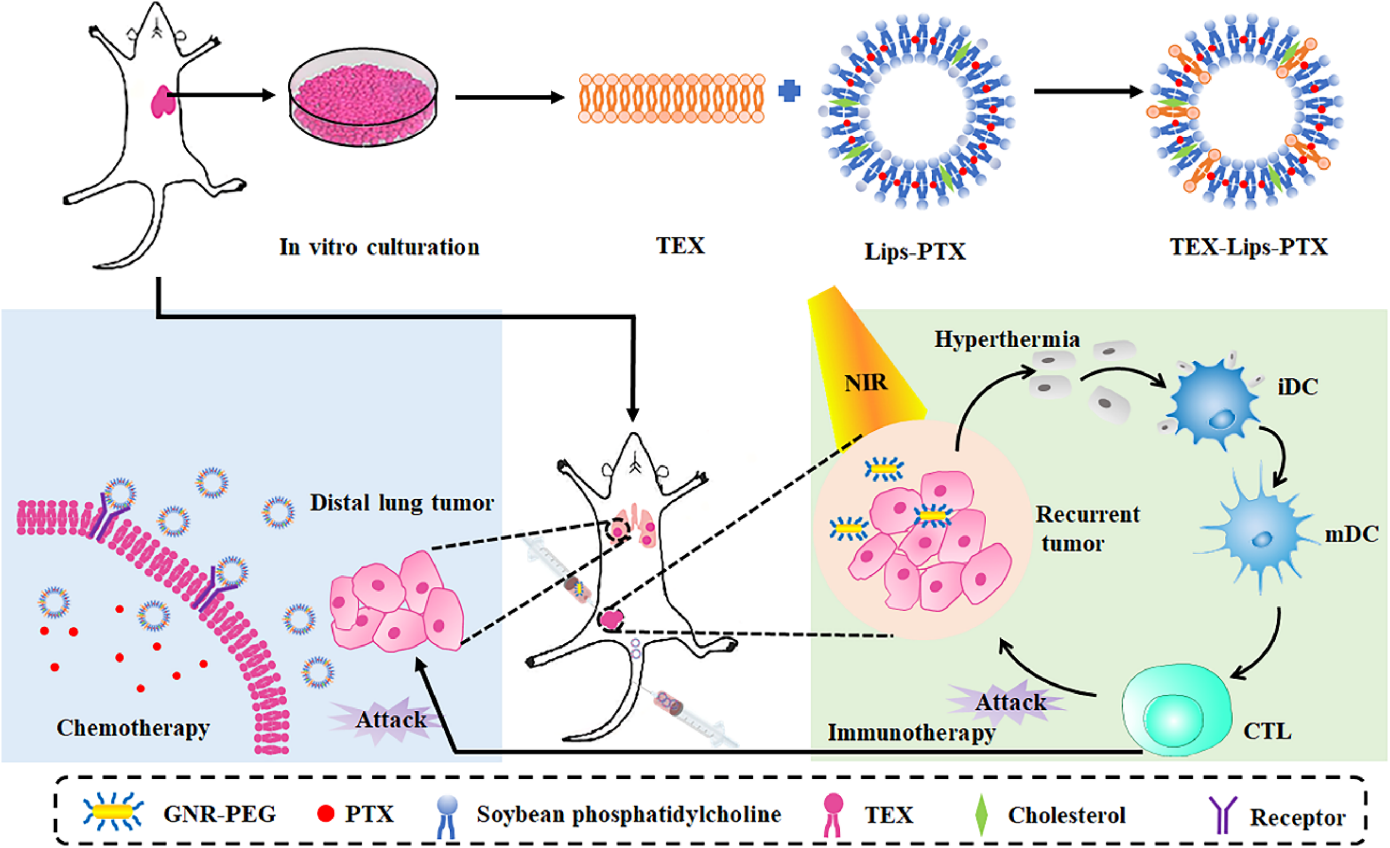
Illustration of the combination strategy of GNR‐PEG and TEX‐Liposome‐PTX to suppress the advanced recurrent and metastatic breast cancer. GNR, gold nanorod; PTX, paclitaxel; TEX, tumor‐derived exosome

Inspired by the above technological progresses, a synergistic strategy based on GNR‐PEG and TEX‐Liposome‐paclitaxel (PTX) is constructed for targeted treatment of recurrent tumors and distant metastases of advanced breast cancer, respectively (Scheme 1). In this delivery system, GNR‐PEG‐mediated hyperthermia served as the pioneer to kill recurrent tumors by thermal ablation via in‐situ NIR irradiation. Meanwhile, the apoptosis of recurrent tumor cells allowed DC to be recruited, and then the tumor antigens were presented to native T cells, stimulating the adaptive immunity via CD8^+^ T cell pathway. Then, TEX membrane with inherent lung homing affinity was hybrid into Liposome‐PTX, forming TEX‐Liposome‐PTX. Sequentially, TEX‐Liposome‐PTX was adopted for targeted chemotherapy of distal lung metastasis tumors by intravenous injection. In addition, the adaptive immune response triggered by hyperthermia ablation exhibited adjuvant therapy for lung metastatic tumors, thereby enhancing the efficacy of chemotherapy, and providing a potential effective targeted therapy strategy for advanced recurrent and metastatic breast cancer in clinical.

## 2 MATERIALS AND METHODS

### 2.1 Materials

Soy Lecithin and cholesterol were obtained from Avanti. PTX was obtained from Aladdin Chemical Co., Ltd. Sulfhydryl polyethylene glycol (SH‐PEG2000) and DSPE‐PEG‐FITC were purchased from Shanghai ToYong Bio Tech. Inc. Chloroauric acid trihydrate (HAuCl_4_·3H_2_O) was purchased from Dibo Biological Technology Co., Ltd. Hexadecyl trimethyl ammonium bromide (CTAB) Shanghai Mokai Biological Technology Co., Ltd. Enhanced BCA Protein Assay Kit, 3‐(4,5‐dimethylthiazol‐2‐yl)‐2,5‐diphenyl‐2H‐tetrazolium bromide (MTT), and penicillin–streptomycin solution were purchased from Beyotime. 4′,6‐diamidino‐2‐phenylindole (DAPI), Dulbecco's modified Eagle medium (DMEM) medium with high glucose, and RPMI1640 medium were purchased from HyClone. 3,3′‐dioctadecyloxacarbocyanine perchlorate (DiO) was obtained from Protein Biological Technology Co., Ltd. (Nanjing, China). Alexa Fluor 647 anti‐CD9 antibody was purchased from Abcam. CD11c‐FITC, CD3‐FITC, CD4‐APC, CD8‐Percp‐Cy5.5, CD80‐APC, and CD86‐PE antibodies were purchased from BD Pharmingen. ELISA kits for interlekin‐6 (IL‐6), interferon‐γ (IFN‐γ), and tumor necrosis factor‐α (TNF‐α) were obtained from Multi Sciences Biotech, Co., Ltd. The other chemical reagents with analytical grade were obtained from Solarbio Life Sciences.

### 2.2 Cells and animals

Murine breast cancer cells (4T1), mouse embryonic fibroblasts cells (NIH 3T3), and human umbilical vein endothelial cells (HUVEC) were obtained from the American Type Culture Collection. BALB/c mice (5–6 weeks, female) were supplied by the experimental animal center of Nantong University. The animal experiments were performed in adherence to the principles expressed in the Declaration of Helsinki, and the experimental protocols were approved by the animal center of Nantong University.

### 2.3 Synthesis and characterization of GNR‐PEG

#### 2.3.1 Synthesis of GNR

Nanorods (GNR) were synthesized on the basis of the seed‐mediated growth method, accompanying some improvements. Briefly, 0.01 M sodium borohydride was adopted to reduce 10 mM chloroauric acid in the presence of 0.1 M CTAB, forming the seed solution. Then, the 0.5 L growth solution containing 0.08 M CTAB, 0.02 M sodium oleate, 24.2 mM chloroauric acid, and 3 mM silver nitrate were prepared. Hydrochloric acid (2.1 ml) and 0.07 mM ascorbic acid were added to acidify the growth solution, and 0.8 ml of the seed solution was introduced into the growth solution subsequently. The solution containing GNR was obtained by standing for 12 h.

#### 2.3.2 Preparation and characterization of GNR‐PEG

The above obtained GNR solution was centrifuged at 10,000 rpm for 20 min and resuspended in water. The 6 mg/ml PEG‐SH solution was introduced into the GNR suspension solution under the condition of vigorous stirring at 50°C. The mixture was stirred for 12 h at 50°C and centrifuged at 10,000 rpm for 20 min. The obtained GNR‐PEG was resuspended in water, cleaned by ultrasonication and centrifuged to remove the CTAB, and unreacted PEG‐SH. GNR‐PEG was prepared successfully and resuspended in ethanol.

Particle size and zeta potentials of GNR‐PEG were evaluated by Malvern Zetasizer Nano ZS90 (Malvern Instruments Ltd.). The morphology of GNR‐PEG was observed by high‐resolution transmission electron microscopy (TEM; JEOL). Absorption spectra of GNR‐PEG was detected by the UV–Vis spectrophotometer (JingHua Technological Instrument Corporation). The photothermal effect of GNR‐PEG was evaluated by the 808 nm NIR laser irradiation (808 nm; LEO‐Photoelectric).

### 2.4 Synthesis and characterization of TEX‐Liposome‐PTX

#### 2.4.1 Preparation of Liposome‐PTX

The thin‐film hydration method was adopted to prepare Liposome‐PTX. Briefly, soy lecithin, cholesterol, and PTX (10:1:1 wt/wt) were dissolved in 15 ml chloroform. Subsequently, chloroform was evaporated to form the thin film under vacuum at 37°C for 2 h and the prepared thin film was further dried under vacuum overnight. Finally, the film was hydrated with 60 ml phosphate‐buffered saline (PBS), stirred at 37°C for 3 h, and downsized by sonication under the condition of 300 W.

#### 2.4.2 Extraction and separation of TEX

The model of 4T1 tumor‐bearing mice was established to acquire the autologous breast cancer cells. The protocol to extract exosome was in accordance with the published article in our laboratory.^34^ Exosome was harvested from the autologous breast cancer cells culture medium by ultracentrifugation. Briefly, the autologous breast cells were cultured for 48 h in FBS‐free high‐glucose DMEM medium. The concomitant culture medium was collected, and a gradient centrifugation was used to isolate the exosome as the following conditions. The supernatant was centrifuged at 305 × *g* for 10 min to dislodge the cells, centrifuged at 2005 × *g* for 15 min to remove cell debris, and centrifuged at 100,000 × *g* for 75 min to isolation exosomes. The exosome pellet was washed with PBS containing PMSF, and again pelleted by centrifugation to purify the exosome. The isolated exosome was resuspended in PBS and feezed at −80°C. All the protocol was conducted at 4°C.

Exosome membrane was isolated by the repeated freezing and thawing. The above obtained exosome was resuspended in cold TM buffer, frozen at −80°C rapidly, and thawed in a water bath at 37°C for 5 min. Repeated freeze–thaw cycle five times in the above conditions. Next, 1 M sucrose was added into the treated exosomes to form a final concentration of 0.25 M, and the mixture was centrifuged at 2000 × *g* for 12 min at 4°C. The collected supernatant was centrifuged again to harvest the exosome membranes. The membranes were resuspended in TM buffer and further centrifugated at 3000 × *g* for 32 min at 4°C.

#### 2.4.3 Preparation and characterization of TEX‐Liposome‐PTX

TEX‐Liposome‐PTX was obtained by incubation and extrusion method. The exosome membranes and Liposome‐PTX were first mixed and incubated overnight under the condition of 500 rpm at 37°C. The mixture of exosome membranes and Liposome‐PTX was further extruded through the extruder (Avanti Polar Lipids) with 0.2 μm pore diameter for fusion. The mass ratio of protein and phospholipid of the prepared exosomes biomimetic liposome was 1:100, 1:20, and 1:5.

Particle size and surface charge of Liposome‐PTX and TEX‐Liposome‐PTX were detected by Malvern Zetasizer (Nano ZS90; Malvern Instruments Ltd.). The morphology of Liposome‐PTX and TEX‐Liposome‐PTX stained with 2% phospotungstic acid was observed by TEM. Sodium dodecyl sulfate–polyacrylamide gel electrophoresis (SDS‐PAGE) was adopted to evaluate the characteristic proteins of 4T1 cells, TEX, TEX membrane, and TEX‐Liposome (1/20). The drug‐loading content and encapsulation efficiency of PTX in Liposome‐PTX and TEX‐Liposome‐PTX were determined by high‐performance liquid chromatography (HPLC; Shimadzu). Meanwhile, the in vitro cumulative release of Liposome‐PTX and TEX‐Liposome‐PTX was conducted by the dialysis method and detected by HPLC.^9^

To identify the successful fusion between TEX membrane and Liposome, CLSM was used to verify the fusion. DSPE‐PEG‐FITC phospholipid was adopted for Liposome preparation. Thus, Liposome was labeled by FITC (green). TEX membrane was incubated with Alexa Fluor 647 anti‐CD9 antibody. Therefore, TEX was indicated by Alexa Fluor 647 (red). Afterwards, the mixture of TEX membrane and Liposome and the hybrid TEX‐Liposome‐PTX were incubated with NIH3T3 cells for 4 h. Next, NIH3T3 cells were fixed with 4% paraformaldehyde and subsequently treated with Hoechst 33342 for 15 min. Finally, the fluorescence was recorded by CLSM and the fusion was proved by the overlap of Alexa Fluor 647 and FITC.

### 2.5 In vitro biocompatibility of GNR‐PEG and TEX‐Liposome‐PTX

MTT assay was conducted to evaluate the biocompatibility of GNR‐PEG and TEX‐Liposome‐PTX. HUVEC cells were seeded at 1.0 × 10^4^ cells/well in a 96‐well plate and incubated to 70%–80% confluence. DMEM medium containing different concentrations of GNR and GNR‐PEG nanoparticles was added into the plate (concentration of GNR 5, 10, 20, 40, and 80 μg/ml) and cells were treated for 24 h. After the wash of PBS for three times, 30 μl MTT solution was added into each well and incubated for 4 h at 37°C. The intracellular formazan crystals were dissolved by DMSO (150 μl). The optical density was detected at 570 nm.

### 2.6 In vitro antitumor assays

#### 2.6.1 Photothermal cytotoxicity of GNR and GNR‐PEG

Photothermal cytotoxicity of GNR and GNR‐PEG on 4T1 cells was evaluated by proliferation inhibition by MTT assay. 4T1 cells were seeded into a 96‐well plate at a density of 9000/well. Subsequently, the medium was replaced by 30 μg/ml GNR or GNR‐PEG and each well was irradiated by 808 nm NIR laser for 1, 2, 4, 8, and 10 min (1.0 W/cm^2^). Afterwards, the medium containing formulations was discarded and substituted by the RPMI1640 medium with FBS. Cells were cultured for 24 h and the cell viability was evaluated by MTT assay.

#### 2.6.2 Cytotoxicity of TEX‐Liposome‐PTX

To assess the treatment effect of TEX‐Liposome‐PTX, MTT assay was performed as the same as the above protocol. After the 4T1 cells were seeded into the 96‐well plates, different concentrations (0.1, 1, 5, 20, 40, and 80 μg/ml) of PTX, Liposome‐PTX, and TEX‐Liposome‐PTX were added and incubated for another 24 h. The cytotoxicity of TEX‐Liposome‐PTX was conducted by MTT assay.

### 2.7 In vitro cellular uptake and targeting of TEX‐Liposome

Cellular uptake of TEX‐Liposome was estimated using CLSM and the cellular targeting ability was evaluated through the different ratio of TEX and Liposome. Briefly, NIH3T3 cells were seeded into the confocal dishes at a density of 1.0 × 10^5^ cells/dish and incubated for 24 h in the incubator. Afterward, the culture medium was discarded and replaced with DiO‐labeled Liposome formulations. The ratio of Liposome and TEX in the various formulations was 1:0, 100:1, 20:1, and 5:1. After incubation for 0.5, 2, and 4 h, the cellular uptake of NIH3T3 cells was stopped with the cold PBS and fixed with 4% paraformaldehyde for 10 min. Subsequently, the nuclei were stained by incubating with DAPI for 10 min at room temperature. The fluorescent images were obtained by CLSM (TCS SP5; Leica) and semi‐quantitative analysis was performed using ImageJ.

### 2.8 In vivo lung‐targeting analysis and biodistribution of TEX‐Liposome

#### 2.8.1 Metastatic breast cancer mouse model (advanced breast cancer mouse model)

4T1 cells (2 × 10^7^ cells/ml) were injected into the right armpit of female BALB/c mice to establish the mice model for investigating the in vivo lung‐targeting. When the solid tumor volume in the right armpit reached 75 mm^3^, the tumor was removed surgically. Subsequently, 4T1 cells were injected into the left armpit and intratail vein respectively when the mice recovered. Finally, the in vivo advanced breast cancer mouse model was constructed successfully.

#### 2.8.2 Lung‐targeting analysis and distribution of TEX‐Liposome

To investigate the premetastatic niche targeting ability of TEX‐Liposome nanoparticles, DiO was used as a fluorescent probe to evaluate the in vivo lung‐targeting ability and distribution of Liposome and TEX‐Liposome (1/100, 1/20, 1/5). DiO, replacing PTX, was encapsulated into TEX‐Liposome according to the liposome manufacturing process in Section 2.4.1. Two hundred microliters formulations (DiO, 50 μg/kg) in the four separate groups were administrated into the above advanced breast cancer model via the tail vein and perfused with saline and 4% paraformaldehyde successively after different times (4, 12, and 24 h). Subsequently, the organs (lung, heart, kidney, liver, and spleen) were collected and fixed in 4% paraformaldehyde for tissue sectioning. The sliced organs were photographed by the CLSM and quantified by Image J.

#### 2.8.3 In vivo imaging analysis of TEX‐Liposome

Furthermore, in vivo imaging was conducted to investigate the lung‐targeting and biodistribution of TEX‐Liposomes in advanced breast cancer mouse model by IVIS Lumina II imaging system (Caliper Life Sciences). Mice were divided into four groups randomly: Liposome, TEX‐Liposome (1/100), TEX‐Liposome (1/20), and TEX‐Liposome (1/5). DiR was selected as the fluorescent probe to label the above formulations. The method of DiR encapsulated into TEX‐Liposome was in accordance with the liposome manufacturing process in Section 2.4.1. Different formulations with DiR at the concentration of 50 μg/kg were administrated via the tail vein. The real‐time NIR fluorescence images of TEX‐Liposomes at 4, 12, and 24 h were recorded to investigate the biodistribution. To further confirm the in vivo tissue distribution of formulations, mice were sacrificed 24‐h postinjection and the harvested tissues (tumor, heart, liver, spleen, lung, and kidney) were imaged.

### 2.9 In vivo efficacy of TEX‐Liposome‐PTX combined with PTT against the advanced breast cancer

First, in vivo imaging was adopted for evaluating the in vivo treatment efficacy. The advanced breast cancer mice model was established as the above protocol by injecting Luc‐4T1 cells and divided into six groups randomly: (1) saline, (2) TEX‐Liposome‐PTX, (3) GNR, (4) GNR combined with PTX, (5) GNR combined with Liposome‐PTX, (6) GNR combined with TEX‐Liposome‐PTX. For GNR‐mediated PTT, GNR was administered by intratumoral multipoint injection (GNR concentration: 30 μg/ml), and then an 808 nm laser (1.0 W/cm^2^) was used to irradiate the tumor site for 15 min, which was recorded as Day 0 (start treatment). After 24‐h treatment of thermal ablation, 200 μl of saline, PTX, Liposome‐PTX, and TEX‐Liposome‐PTX were injected every other day for a total of four times (PTX dosage: 10 mg/kg). The tumor volume and the body weight were detected every other day. After 12 days of treatment, the tumor‐bearing mice in each group were subjected to bioluminescence in vivo imaging to evaluate the progress of solid tumor metastases on the left side and lung metastases. Afterward, the mice were sacrificed, and the lung was collected, fixed with 4% paraformaldehyde, and then sliced for hematoxylin and eosin (H&E) staining.

### 2.10 Study on the immune regulation mechanism of TEX‐Liposome‐PTX combined with PTT

The in vivo advanced breast cancer mouse model was constructed for the investigation of immune regulation mechanism of thermal therapy combined with chemotherapy.

#### 2.10.1 Maturation level detection of DC from inguinal lymph nodes

To explore the immunological mechanism of photothermal combined with chemotherapy for the treatment of advanced breast cancer, maturation level of DC in the left axillary lymph nodes was detected after a single dose of PTX‐chemotherapy according to the protocol.^36^ DC cells were incubated with CD11c‐FITC, CD80‐APC, and CD86‐PE antibodies for 20 min at 4°C and detected by flow cytometry (FCM).

#### 2.10.2 Detection of T lymphocyte subsets from lung tissue

To investigate T cells immune responses, lung was harvested after a single dose of PTX‐chemotherapy. After separating from each group, T lymphocyte was stained with CD3‐FITC, CD8‐Percp‐Cy5.5, and CD4‐APC antibodies by incubating for 20 min at 4°C. The T lymphocyte differentiation in lung at different groups were detected by FCM.

#### 2.10.3 Detection of cytokine levels

After being treated with a single dose of PTX‐chemotherapy, the peripheral blood of mice was taken and coagulated at room temperature for 15 min. Then, samples were centrifuged at 3000 rpm for 15 min, and the serum was obtained. The concentration of TNF‐α, IFN‐γ, and IL‐6 in serum was measured according to the manufacture's operating protocol in the ELISA reagent test kit.

### 2.11 Statistical analysis

The above‐mentioned data were processed by GraphPad Prism software (version 8.0) and expressed as mean ± *SD*. The difference between the two independent groups was performed by a Student's *t*‐test. *p* < 0.05 was considered statistical significance.

## 3 RESULTS AND DISCUSSION

### 3.1 Preparation and characterization of GNR‐PEG

**FIGURE 1:**
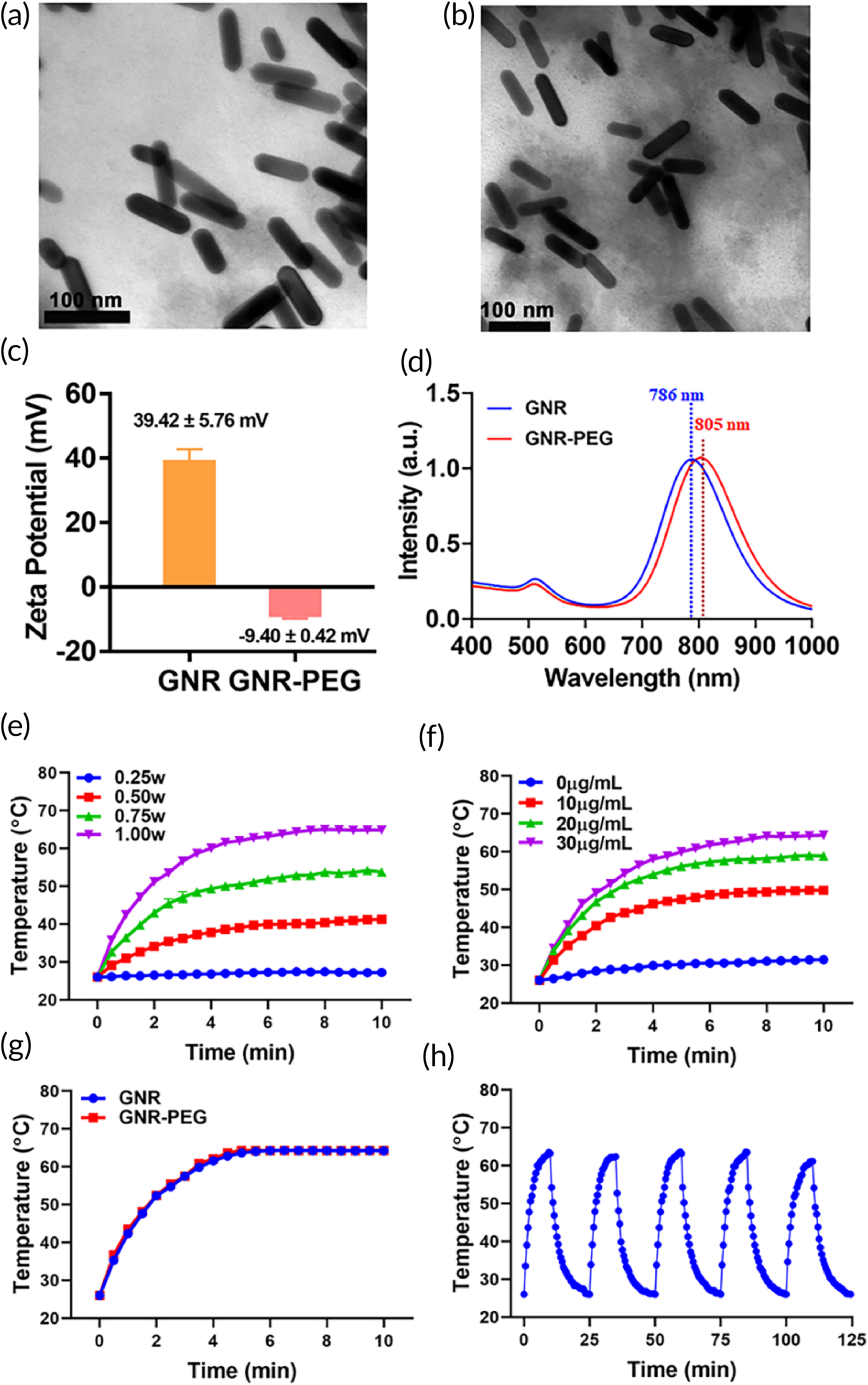
The characterization of GNR and GNR‐PEG. TEM images of GNR (a) and GNR‐PEG (b). (c) Zeta potential of GNR and GNR‐PEG detected by Malvern Nano ZA90 (*n* = 3). (d) The UV‐spectroscopy of GNR and GNR‐PEG nanoparticles detected by UV–Vis–NIR. (e) The photothermal conversion curves of GNR‐PEG (30 μg/ml) with 808 nm laser irradiation at 0.25, 0.50, 0.75, 1.00 W (*n* = 3). (f) The photothermal conversion curves of GNR‐PEG with different concentrations after 808 nm laser irradiation at 1.00 W/cm^2^ (*n* = 3). (g) The temperature variation curves of GNR and GNR‐PEG (GNR, 30 μg/ml) with 808 nm laser irradiation at 1.00 W/cm^2^ (*n* = 3). (h) Photothermal stability of 30 μg/ml GNR‐PEG for five on and off cycles with 808 nm laser irradiation at 1.00 W/cm^2^. GNR, gold nanorod; TEM, transmission electron microscopy; UV, ultraviolet

GNR‐PEG as an effective PTT agent was synthesized according to the seed growth method. To surmount the aggregation instability and cell cytotoxicity of GNR, PEG was modified on the surface. As presented in TEM images (Figure 1a,b), both GNR and GNR‐PEG were rod‐shaped structures with circular arcs at both ends, with a long diameter of 82 nm and a width diameter of 21 nm. After surface modification with PEG, the rod‐shaped structure of GNR‐PEG kept the original shape of GNR with relatively uniform dispersion. The zeta potential of GNR and GNR‐PEG was exhibited in Figure 1c. Surface modification of PEG reversed the zeta potential of GNR from 39.42 ± 5.76 to −9.40 ± 0.42 mV, validating the successful surface decoration of PEG on the GNR surface. The surface charge of GNR‐PEG reversed from positive to negative, might imply that the cell cytotoxicity could be reduced. In the ultraviolet (UV)–visible spectrum (Figure 1d), 786 and 805 nm were the characteristic absorption peaks of GNR and GNR‐PEG, respectively. After surface modification with PEG, the ultraviolet absorption peak had a red‐shifted, resulting in an elevated photothermal conversion ability of GNR‐PEG under NIR irradiation at 808 nm.

Moreover, the photothermal conversion effect of GNR‐PEG was investigated to confirm the thermal response capability triggered by 808 nm NIR laser. The GNR‐PEG exhibited irradiation intensity dependence and concentration dependence photothermal effect, presenting a robust photothermal conversion capability (Figure 1e,f). To perform effective PTT, the intensity of 1.0 W/cm^2^ was selected in the subsequent animal studies. According to Figure 1g, the temperature variation curves of GNR negligibly changed after PEG decorated. Finally, the photothermal conversion stability of GNR‐PEG was investigated under five on and off cycles with the irradiation of 808 nm. As exhibited in Figure 1h, the stable photothermal conversion capability proved the good stability of GNR‐PEG nanoparticles.

### 3.2 Preparation and characterization of biomimetic TEX‐Liposome‐PTX

**FIGURE 2:**
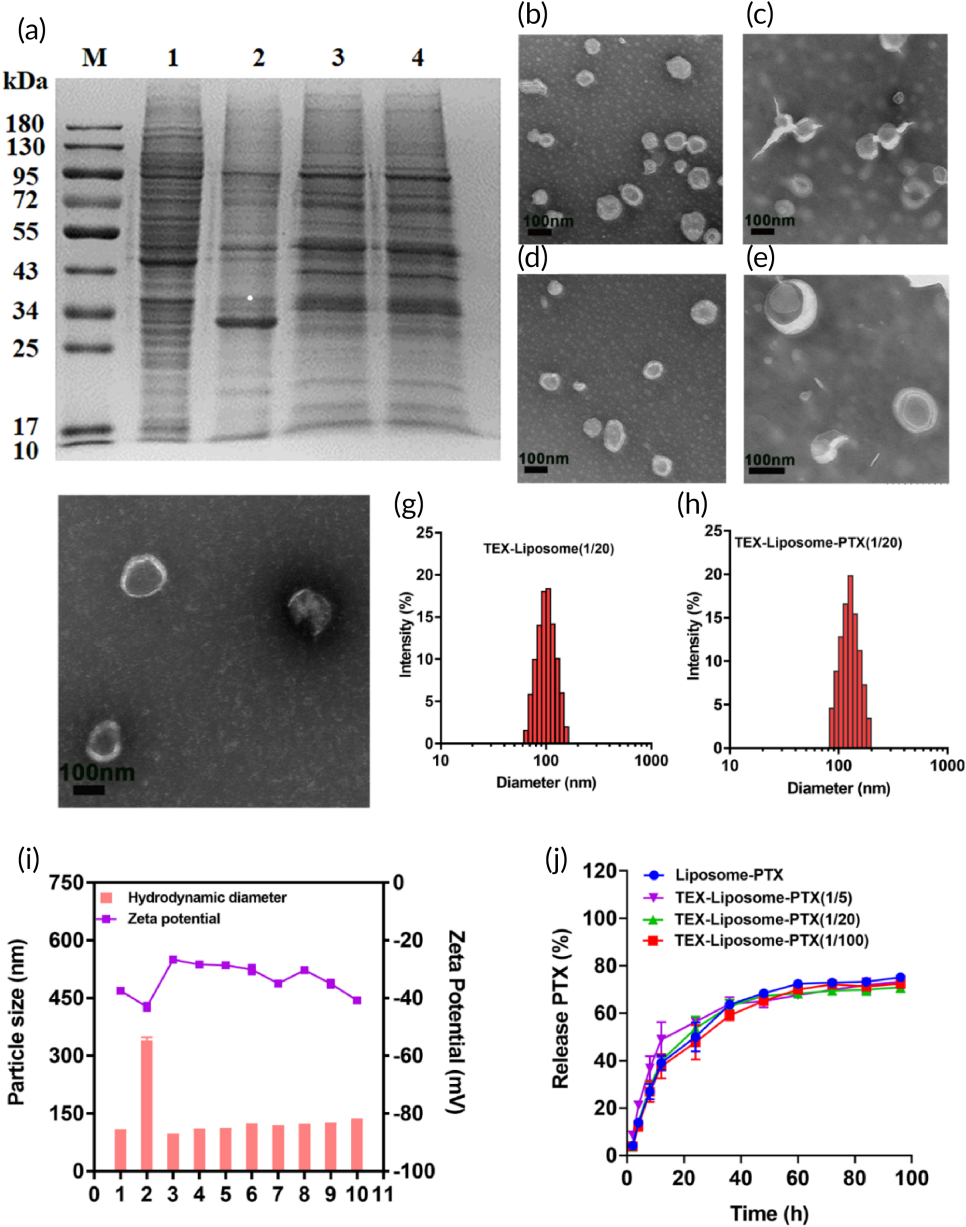
(a) SDS‐PAGE image for protein analysis of (1) 4T1 cells, (2) TEX, (3) TEX‐membrane, and (4) TEX‐Liposome (1/20). TEM images of exosome (b), Liposome (C), Liposome‐PTX (D), TEX‐Liposome (1/20) (E), and TEX‐Liposome‐PTX (1/20) (F) with negative staining with 2% phosphotungstic acid (scale bar = 100 nm). Particle size distribution of TEX‐Liposome (1/20) (G) and TEX‐Liposome‐PTX (1/20) (H) detected by DLS. (I) The hydrodynamic diameters and zeta potentials of different nanoparticles (1, Exosome; 2, Exosome membrane; 3, Liposome; 4, TEX‐Liposome (1/100); 5, TEX‐Liposome (1/20); 6, TEX‐Liposome (1/5); 7, Liposome‐PTX; 8,TEX‐Liposome‐PTX (1/100); 9, TEX‐Liposome‐PTX (1/20); 10, TEX‐Liposome‐PTX (1/5)). (J) The in vitro release profiles of PTX in different nanoparticles (*n* = 3). DLS, dynamic light scattering; PTX, paclitaxel; SDS‐PAGE, sodium dodecyl sulfate–polyacrylamide gel; TEX, tumor‐derived exosome; TEM, transmission electron microscopy

As previously reported, the PTX‐loaded liposomes were prepared by thin‐film hydration method. The hydrodynamic size and zeta potential of Liposome‐PTX were measured by dynamic light scattering (DLS). After PTX loaded, an increased size of 20 nm and a decreased zeta potential of 9 mV were observed (Table S1). The morphology of Liposome‐PTX was visualized by TEM with negative staining. Both Liposomes and Liposome‐PTX presented obvious unilamellar vesicle morphology observed in Figure 2c,d, and the sizes were consistent with hydrodynamic size detected by DLS. Drug loading and encapsulation efficiency detected by HPLC were 9.99 ± 0.96% and 97.58 ± 1.36%, respectively. The dialysis method was adopted to investigate the in vitro release behavior of PTX‐loaded liposomes in PBS solution. As exhibited in Figure 2j, PTX released from liposome slowly, reaching 70% within 100 h and presenting sustained release behavior.

**FIGURE 3:**
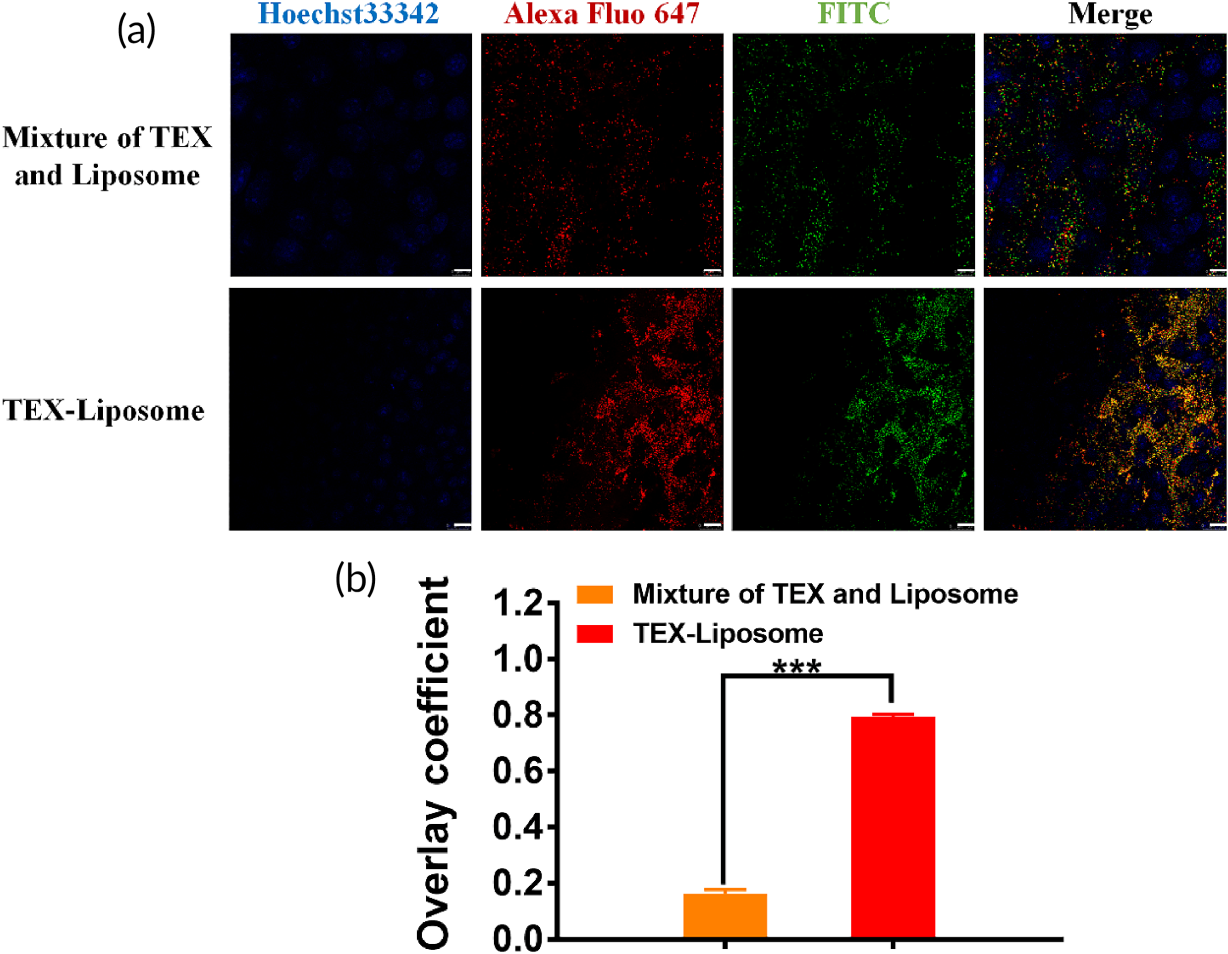
Verification of TEX‐Liposome generation. (a) CLSM images of cellular uptake of untreated mixture and TEX‐Liposome nanoparticles after incubation with NIH3T3 cells for 4 h. Green for FITC labeled Liposome which was prepared by DSPE‐PEG‐FITC phospholipid. Red for Alexa Fluor 647‐anti‐CD9 (TEX specific membrane protein) antibody labeled TEX membrane. The nucleus was indicated using Hoechst 33342 (Blue). Scale bars = 10 μm. (b) The overlay coefficient analysis according to (a) using Image J. ***p < 0.001. TEX, tumor‐derived exosome

To enable lung‐PMN targeting of Liposome‐PTX, exosome membranes were extracted from autologous breast cancer cells using ultracentrifugation and repeated freezing and thawing, fused into Liposome by incubation–extrusion method, forming an exosome hybrid liposome delivery system.^37^ The harvested exosomes were characterized by DLS and TEM (Figure 2b and Table S1). The particle size of exosome was 106.63 ± 1.24 nm, and the zeta potential was −37.45 ± 1.53 mV. A round‐shape morphology with the visible lipid layer of exosomes was observed by TEM, which was consistent with the previous literature,^34^ demonstrating the successful extraction of exosomes. Furthermore, according to intuitive evidence from Figure 3, the overlay coefficient of Alexa Fluor 647 (red) and FITC (green) in TEX‐Liposome was significantly different from those in the mixture, indicating the successful fusion of TEX membrane and Liposome. After fusion with Liposome, SDS‐PAGE was conducted (Figure 2a). Compared with exosome, the protein component of exosome membrane was slightly reduced due to the removal of intracellular proteins. While the protein analysis of biomimetic TEX‐Liposome retained the characteristic protein and exhibited negligible difference with TEX membrane, indicating the successful fusion. Moreover, the detailed morphology of Liposome‐TEX (Figure 2e,f) still maintained the round‐shape with visible lipid layer and well dispersion, further suggesting that the exosome membrane could hybridize into the liposome membrane successfully. Similarly, PDI of the TEX‐Liposome nanoparticles detected by DLS was less than 0.3 (Table S1), and the particle size distribution exhibited in Figure 2g,h proved the better dispersion properties of the hybrid nanoparticles again. As presented in Table S1, the particle size of exosome membrane was 336.47 ± 20.33 nm, greater than exosome. With the proportion of exosome membranes increasing in the hybrid nanoparticles and the loading of PTX, the particle size increased gradually (Figure 2i and Table S1). Finally, the successful hybridization of exosome membrane and liposomes could also be confirmed by surface potential. The zeta potentials of hybrid nanoparticles prepared in all proportions were between the exosome membrane and liposomes, demonstrating the successful manufacture of hybrid nanoparticles.

The encapsulation efficiency of TEX‐Liposome‐PTX with various proportions was ~97%, and PTX contents were all above 9.2%, showing negligibly significant difference with that of Liposome‐PTX (Table 1). Meanwhile, the in vitro cumulative release behavior of TEX‐Liposome‐PTX with different proportions was investigated, displaying a sustained release, similar to the behavior of Liposome‐PTX.

**TABLE 1:** Drug loading and encapsulation efficiency detected by HPLC (*n* = 3)

Samples	Drug loading (%)	Encapsulation efficiency (%)
Liposome‐PTX	9.99 ± 0.96	97.58 ± 1.36
TEX‐Liposome‐PTX (1/100)	9.29 ± 0.03	97.04 ± 0.66
TEX‐Liposome‐PTX (1/20)	9.54 ± 0.03	97.45 ± 0.25
TEX‐Liposome‐PTX (1/5)	9.73 ± 0.05	97.71 ± 0.26

Abbreviations: HPLC, high‐performance liquid chromatography; PTX, paclitaxel; TEX, tumor‐derived exosome.

### 3.3 In vitro biocompatibility of GNR‐PEG and TEX‐Liposome‐PTX

**FIGURE 4:**
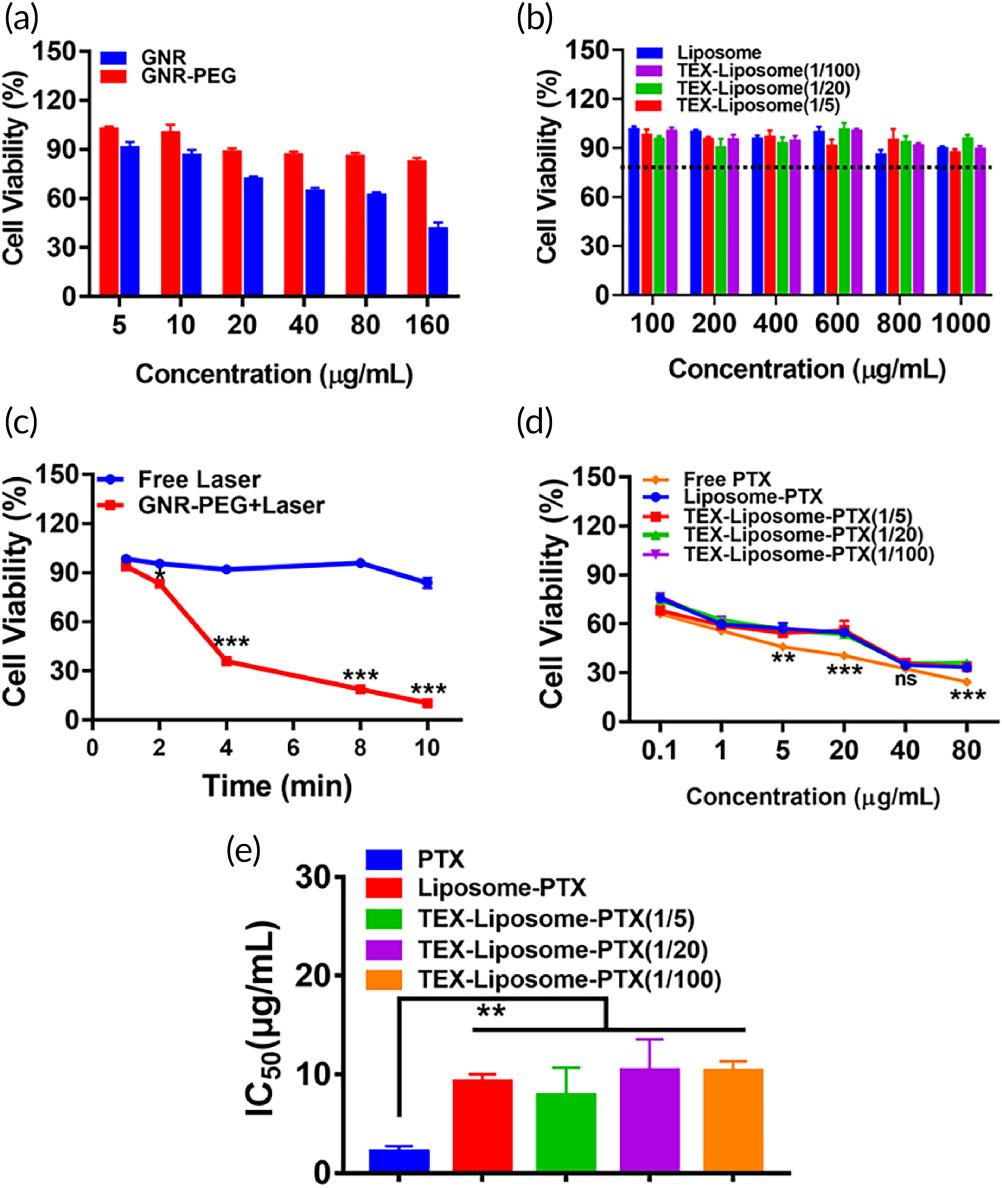
(a) Viability of HUVEC cells treated with different concentration of GNR and GNR‐PEG following incubation for 24 h. (b) Cell viability of HUVEC treated with Liposome, TEX‐Liposome (1/5), TEX‐Liposome (1/20), and TEX‐Liposome (1/100). (c) Viability of 4T1 cells following irradiation of 808 nm NIR laser at 1.00 W/cm^2^ for different time periods. ***p < 0.001 versus free laser. (d) Viability of 4T1 cells treated with PTX, Liposome‐PTX, TEX‐Liposome‐PTX (1/5), TEX‐Liposome‐PTX (1/20), TEX‐Liposome‐PTX (1/100) with different concentration of PTX following incubation for 24 h. **p < 0.01, ***p < 0.001 versus PTX. (e) IC_50_ values of PTX, Liposome‐PTX, TEX‐Liposome‐PTX (1/5), TEX‐Liposome‐PTX (1/20), and TEX‐Liposome‐PTX (1/100) on 4T1 cells. **p < 0.01 versus PTX. GNR, gold nanorod; HUVEC, human umbilical vein endothelial cell; PTX, paclitaxel; TEX, tumor‐derived exosome

Cytotoxicity of delivery nanosystem limits the in vivo application. Therefore, an excellent vehicle should have satisfactory biocompatibility. As reported, TEX have inherent affinity to interact with endothelial cells.^38^ Thus, we chose HUVEC cells to investigate the biocompatibility of GNR‐PEG by MTT assay. As exhibited in Figure 3a, cell viability was more than 80% after being treated with GNR‐PEG at the concentration range of 5–160 μg/ml, displaying excellent biocompatibility. Nevertheless, with the concentration up to 80 μg/ml, cell viability of HUVEC treated with GNR was approximately 60%, suggesting obvious cytotoxicity of positively charged GNR. The half‐maximum inhibitory concentration (IC_50_) of GNR and GNR‐PEG in HUVEC cells was calculated to be 119.2 and 262.9 μg/ml, respectively. Distinctly, the surface decoration of PEG‐reduced cytotoxicity by reversing the surface charge of GNR. Next, the cell viability of Liposome and TEX‐Liposome was detected against HUVEC cells. As shown in Figure 4b, Liposome and TEX‐Liposome with various proportions presented negligible cytotoxicity towards HUVEC cells, verifying the high biocompatibility.

### 3.4 In vitro cellular uptake and targeting of TEX‐Liposome

**FIGURE 5:**
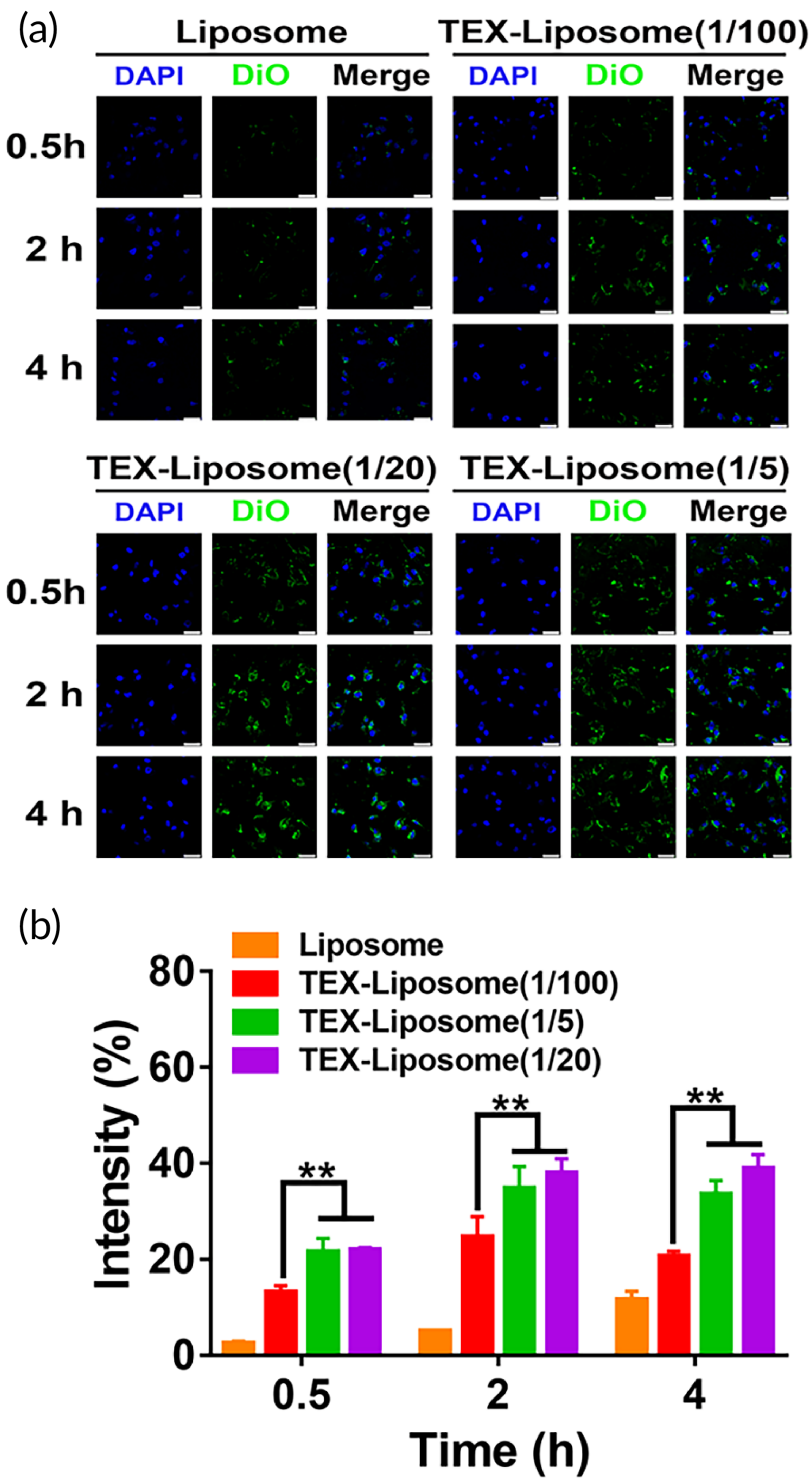
Cellular uptake of TEX‐Liposome in NIH3T3 cells. (a) In vitro cellular uptake of Liposome and TEX‐Liposome‐PTX (1/5, 1/20, 1/100) observed by CLSM at 0.5, 2, and 4 h (scale bar = 25 μm). (b) Quantified analysis of DiO intensity according to (a) by ImageJ. **p < 0.01. DiO, 3,3′‐dioctadecyloxacarbocyanine perchlorate; TEX, tumor‐derived exosome

Pulmonary metastasis is a common phenomenon in advanced breast cancer. As reported, exosomes derived from breast cancer cells have lung homing capability due to the presence of integrins.^29^ Thus, we speculated that TEX hybrid liposome had the lung targeting capability. To evaluate the lung targeting ability, NIH3T3 cells were selected and cell internalization of DiO‐labeled liposomes was monitored by CLSM. As shown in Figure 5a,b, the intracellular fluorescence intensity of DiO presented a time‐dependent enhancement in all treated groups, with the maximum fluorescence intensity at 4 h. The intracellular fluorescence of Liposome presented a significant decrease compared to TEX‐Liposome at each selected time point (0.5, 2, and 4 h). Clearly, the elevated DiO intracellular fluorescence in TEX‐Liposome suggested the high affinity of TEX to NIH3T3 mouse embryonic lung fibroblast cells, which could facilitate the subsequent endocytosis mediated by receptor. Furthermore, the intracellular DiO intensity was augmented with the proportion of exosome in TEX‐Liposome. Compared with TEX‐Liposome‐PTX (1/5) and TEX‐Liposome‐PTX (1/20), the DiO intensity was lower in TEX‐Liposome‐PTX (1/100), suggesting the proportionally lower cellular uptake induced by the lowest exosome proportion. However, the internalization intensity of TEX‐Liposome‐PTX (1/5) and TEX‐Liposome‐PTX (1/20) exhibited no significant difference, with twofold higher than that of TEX‐Liposome‐PTX (1/100). Accordingly, the formed TEX‐Liposome had higher affinity with the target cells when the hybrid proportion of exosome membrane reached over 1/20. Thus, the lung cell target accumulation of TEX‐Liposome could be achieved by receptor‐mediated endocytosis based on integrins.

### 3.5 In vitro antitumor assays

Cellular internalization is the prerequisite for the subsequent antitumor effect. Next, the antiproliferation against 4T1 cells was assessed to evaluate the thermal ablation efficacy induced by GNR‐PEG (Figure 3c). In the absence of GNR‐PEG nanoparticles, the cytotoxicity caused by free NIR laser was negligible within 10 min. Nevertheless, the antiproliferation against 4T1 cells of GNR‐PEG at the concentration of 30 μg/ml presented time‐dependent under NIR irradiation. Cell viability decreased drastically with the extend of irradiation time, exhibiting a very significant difference compared with free laser (*p* < 0.001). With the irradiation time extended to 10 min, cell viability value was about 15%, suggesting a robust photothermal cytotoxicity. In all, in vitro antiproliferation effect of GNR‐PEG detected by MTT verified that GNR‐PEG could be an effective photothermal therapeutic agent with excellent tumor‐killing capacity.

Meanwhile, MTT was employed to examine the in vitro antitumor effect of PTX‐loaded liposome nanoparticles. As shown in Figure 3d, a remarkable concentration‐dependent cytotoxicity on 4T1 cell was observed in all treated groups. Obviously, no significant difference in cytotoxicity was observed between TEX‐Liposome‐PTX and Liposome‐PTX, which was consistent with the previous in vitro cumulative release, and further proved that the hybridization of TEX into liposome could not affect the release behavior from liposome. Nevertheless, free PTX exhibited enhanced cytotoxicity compared with Liposome‐PTX and TEX‐Liposome‐PTX (1/5, 1/20, 1/100), with IC_50_ value decreasing ~3.4‐fold (Table S2 and Figure 3e). The shrinking cytotoxicity in TEX‐Liposome‐PTX and Liposome‐PTX groups might be caused by the presence of liposome delivery system, which might induce the reduction of PTX toxicity in vivo. In all, the designed exosome hybrid liposome nanoparticles could be an effective vehicle for PTX delivery in vivo.

### 3.6 In vivo lung‐targeting analysis and biodistribution of TEX‐Liposome

#### 3.6.1 In vivo lung‐targeting analysis and distribution

**FIGURE 6:**
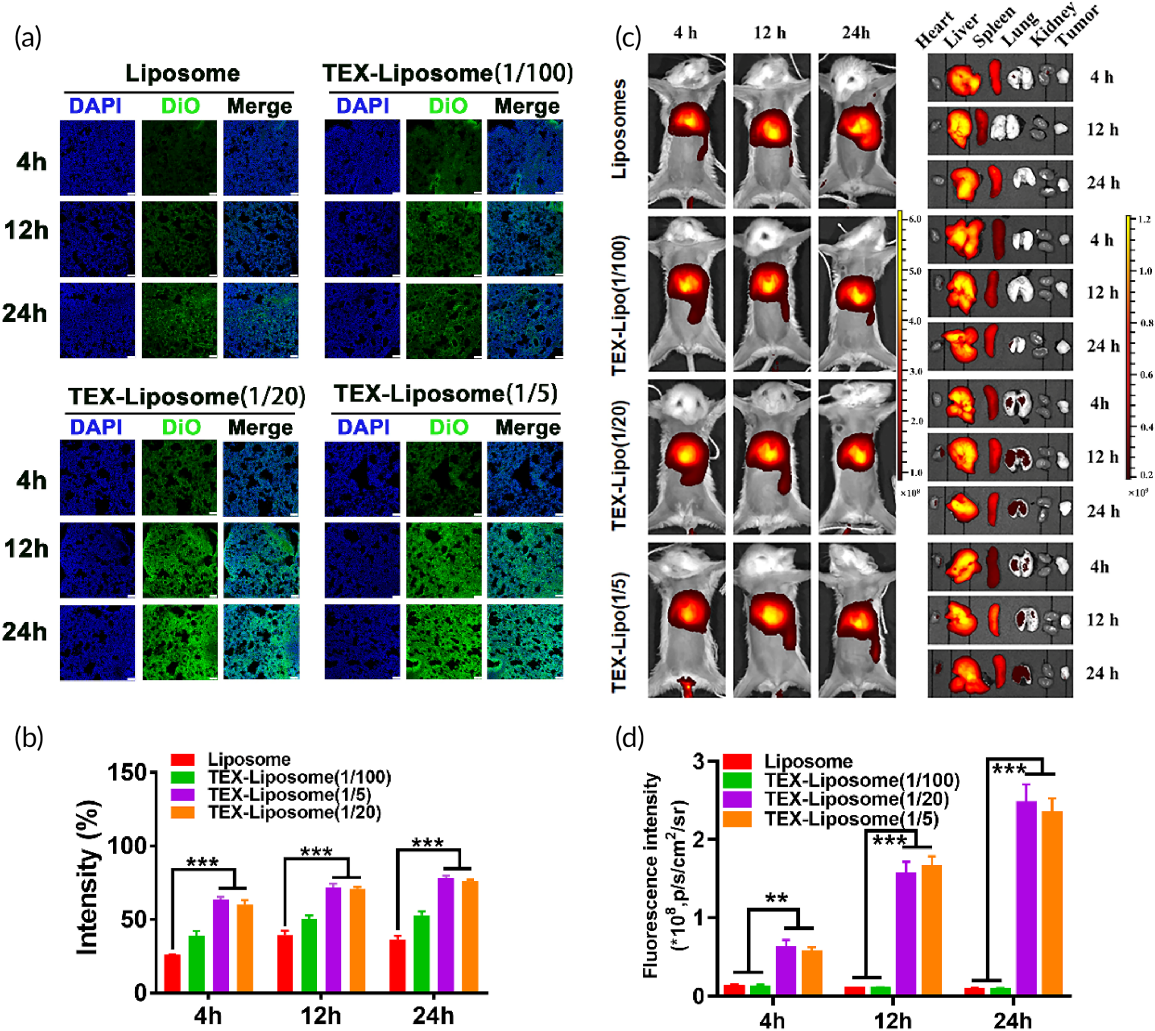
(A) In vivo lung distribution analysis of Liposome, TEX‐Liposome‐PTX (1/5), TEX‐Liposome‐PTX (1/20), and TEX‐Liposome‐PTX (1/100) at 4, 12, and 24 h postinjection observed by CLSM (scale bar of [a] = 75 μm). (b) Quantified analysis of the DiO intensity according to (a). ***p < 0.001. (c) In vivo IVIS spectrum images and biodistribution of Liposome, TEX‐Liposome (1/5), TEX‐Liposome (1/20), and TEX‐Liposome (1/100). (d) Quantification analysis of Liposome, TEX‐Liposome (1/5), TEX‐Liposome (1/20), and TEX‐Liposome (1/100) in lung at different times according to (c). **p < 0.01, ***p < 0.001. DiO, 3,3′‐dioctadecyloxacarbocyanine perchlorate; PTX, paclitaxel; TEX, tumor‐derived exosome

To investigate the lung‐targeting ability and biodistribution of TEX‐Liposome in vivo, an advanced breast cancer model in BALB/c was established. The fluorescent lipid probe DiO was selected to localize the liposomes in vivo. As exhibited in the lung slices (Figure 6a,b), DiO fluorescence intensity of all treated groups increased with time prolonged, reaching highest level at 24 h. It was worth noting that DiO fluorescence intensity in lung treated with TEX‐Liposome (1/100) had a negligible difference with Liposome, exhibiting poor lung targeting capability (*p* < 0.01). While DiO intensity of TEX‐Liposome (1/20) group was similar to that of TEX‐Liposome (1/5) group, demonstrating the specific tropism capability of TEX to lungs. When the proportion of exosome membrane hybrid in liposome was more than 1/20, the TEX‐Liposome was the owner of excellent pulmonary targeting and adhesion capability, which was consistent with the above results of targeting analysis in vitro. Overall, by means of the TEX hybridization, a greater number of TEX‐Liposomes could accumulate in lung. Since there was no significant difference between TEX‐Liposome (1/5) and TEX‐Liposome (1/20) groups in terms of in vitro release, in vivo lung targeting and in vitro cytotoxicity, TEX‐Liposome (1/20) was selected as the therapeutic agent for the follow‐up test.

The accumulation of DiO‐labeled TEX‐Liposome (1/20) in other organs (heart, liver, spleen, kidney, and tumor) was measured and quantified (Figure S1). As observed, there was the strongest fluorescence intensity in liver, indicating the liver accumulation of TEX‐Liposome (1/20). This might be due to the RES effect of the liver.

However, the pulmonary DiO signals were still strong. Notably, only a small amount of TEX‐Liposomes accumulated in tumors. The lower fluorescence intensity in tumors might be due to the lack of affinity between the designed TEX‐Liposome and tumor cells, and the accumulation only relied on the EPR passive targeting.

These results clearly indicated that TEX hybrid liposomes had the advantage of accumulating in pulmonary.

#### 3.6.2 In vivo imaging

To further evaluate the lung targeting ability and in vivo biodistribution of TEX‐Liposome nanoparticles, the advanced breast cancer mice model was constructed to perform in vivo imaging analysis. DiR labeled Liposome and TEX‐Liposome nanoparticles were injected by tail vein and imaged using the IVIS system. As shown in Figure 5c, in vivo dynamic biodistribution process of Liposome and TEX‐Liposome in model mice was recorded at 4‐, 12‐, and 24‐h postinjection. Meanwhile, anatomical data from the mice at selected time points were recorded simultaneously to evaluate specific PMN targeting and biodistribution in vivo (Figure 5c). As exhibited, Liposome was mainly distributed in liver and kidney tissues, and almost no DiR fluorescence was observed in lungs and tumors. A possible explanation was that Liposome, without actively targeting capability, is difficult to reach the lesion. Most Liposomes are eliminated by metabolic liver and kidney organs. Similarly, TEX‐Liposome (1/100) exhibited the analogous biodistribution with Liposome, indicating that TEX‐Liposome (1/100) had no lung targeting capability when the hybridization ratio of TEX is 1/100. In contrast, TEX‐Liposome (1/20 and 1/5) were mainly distributed in liver, kidney, and lung tissues. Notably, no significant difference in DiR fluorescence intensity was observed between the two proportions (Figure 5d). The enhanced accumulation of TEX‐Liposome (1/20 and 1/5) in lung might be due to the inherent lung homing ability of TEX.^34^ More importantly, the DiR signal intensity of TEX‐Liposome (1/20, 1/5) in lung tissue increased with the time extended, which further demonstrated that TEX‐hybrid Liposome had excellent integrin‐mediated lung targeting ability.

### 3.7 In vivo efficacy of TEX‐Liposome‐PTX combined with PTT against the advanced breast cancer

**FIGURE 7:**
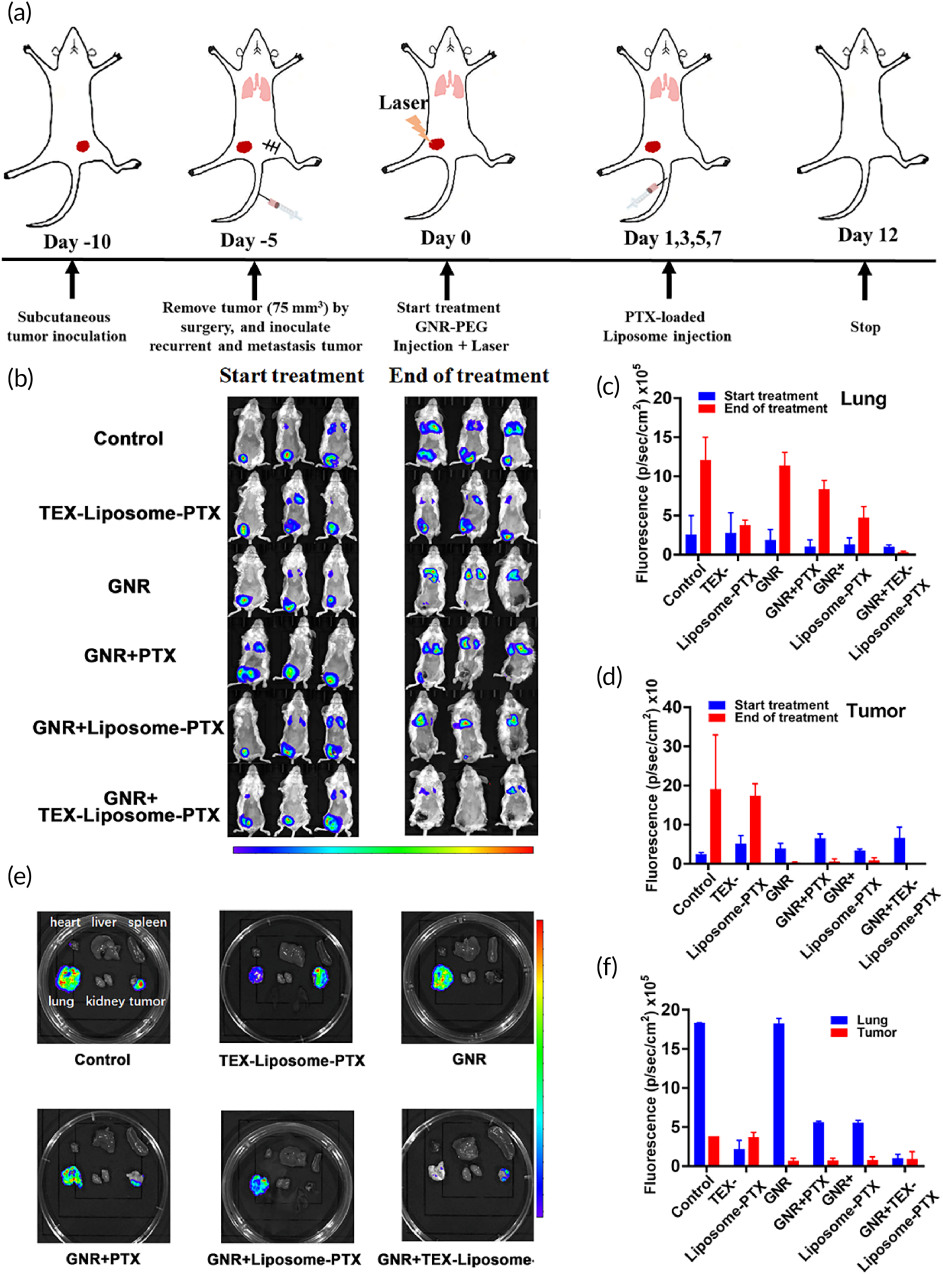
In vivo antitumor effect investigated by bioluminescence imaging in advanced breast cancer mouse model. (a) Schematic illustrating the establish of model and the dosing process. (b) In vivo bioluminescence imaging of mice before and after treatment with different formulation groups. (c) The quantitative fluorescence intensity of lung according to (b). (d) The quantitative fluorescence intensity of tumors according to (b). (e) Bioluminescence images of ex vivo organs in different groups (lung, heart, liver, spleen, kidney, and tumor). (f) The quantitative fluorescence intensity of tumors and lung according to (e)

Results of in vitro antiproliferation against 4T1 cells revealed the better cell inhibitory effects of both GNR and TEX‐Liposome‐PTX. Thus, to evaluate the therapy efficacy of GNR combined with TEX‐Liposome‐PTX in the treatment of advanced breast cancer, luc‐4T1 was selected to construct the mice model (Figure 7a). When the recurrence tumor volume reached 100 mm^3^, the mice were randomly divided into the following six groups: control (saline), TEX‐Liposome‐PTX, GNR, GNR + PTX, GNR + Liposome‐PTX, and GNR + TEX‐Liposome‐PTX. As shown in Figure 7b, bioluminescence imaging was conducted to evaluate whether the model was constructed successfully. At Day 0, fluorescence appeared in lungs and tumors of mice in each group, indicating that the advanced breast cancer mouse model was successfully constructed (Figure 7c,d). Subsequently, GNR, administrated by in situ intratumor injection, was mainly designed for the treatment of recurrent tumors. While TEX‐Liposome‐PTX with lung homing affinity was adopted to treat lung metastases by tail vein injection. The detailed administration strategy was presented in Figure 7a.

After 12 days of therapy with different therapeutic agents, fluorescein substrate was injected via intraperitoneal to evaluate the therapy efficacy through the living bioluminescence imaging (Figure 7b–d). Compared to the control group, the fluorescence intensity in the group treated with TEX‐Liposome‐PTX substantially decreased in lung tissue (***p < 0.001), while having no significant difference in recurrent tumors. This may indicate that TEX‐Liposome‐PTX has a targeted therapeutic effect on lung metastases tumors, proving the excellent lung targeting capability of TEX. For the group treated with GNR, the fluorescence intensity of recurrent tumors was greatly reduced, while there was only a modest reduction of lung tissues, demonstrating the excellent hyperthermia ablation of GNR. Unsurprisingly, the fluorescence of lung metastatic tumors and recurrent tumors both greatly reduced after being treated with the combination strategy of GNR and TEX‐Liposome‐PTX, suggesting the excellent therapeutic effect on advanced breast cancer in vivo. Meanwhile, bioluminescence imaging of isolated organs was measured to further evaluate the therapeutic effect after treatment (Figure 7e,f). As expected, results were consistent with in vivo living bioluminescence imaging, and the combination therapy of GNR and TEX‐Liposome‐PTX presented excellent therapeutic efficacy. These results were consistent with the above design. Specifically, TEX‐Liposome‐PTX possessed a satisfactory targeted therapy effect on lung metastatic tumors, while GNR had an excellent treatment of recurrent solid tumors. Notably, a partial inhibitory effect on lung metastases was observed in groups treated with GNR, might be caused by the adaptive immune response triggered by the thermal ablation of GNR.

**FIGURE 8:**
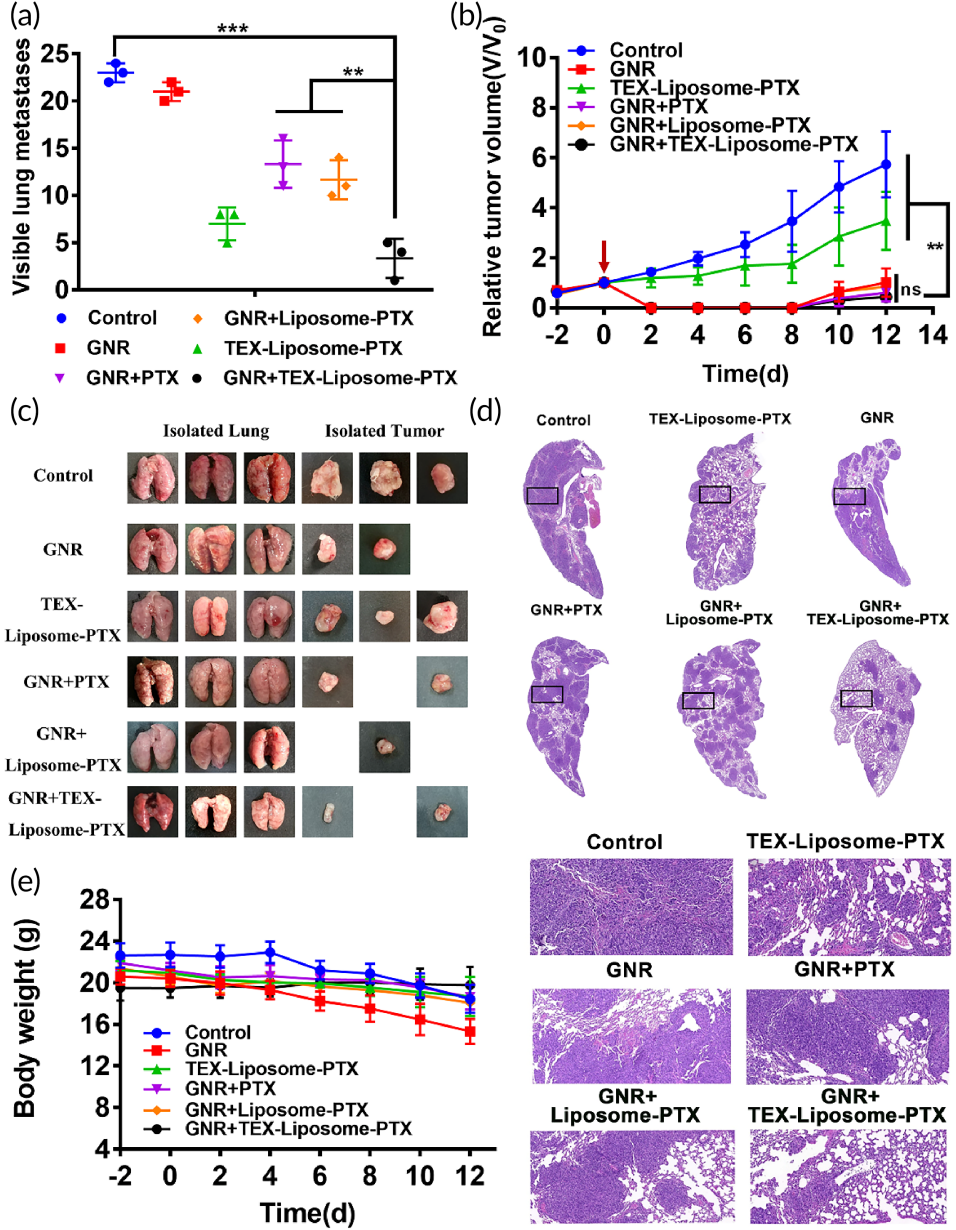
Evaluation of in vivo therapeutic efficacy. (a) Numbers of visible metastatic nodules in each group (*n* = 3). (b) The relative tumor volume curves after treated with different groups (*n* = 3). (c) Images of lung and recurrent tumor isolated from mice treated with saline, TEX‐Liposome‐PTX, GNR, GNR + PTX, GNR + Liposome‐PTX, and GNR + TEX‐Liposome‐PTX. (d) H&E staining images of lung tissues isolated from mice after treatment. (e) Body weight changes of mice recorded every other day during the period of treatment. **p < 0.01, ***p < 0.001. GNR, gold nanorod; H&E, hematoxylin and eosin; PTX, paclitaxel; TEX, tumor‐derived exosome

To further evaluate the lung metastasis and recurrent solid tumors, lung and tumors were collected. A large number of visible metastatic lung nodules were found in control and GNR‐treated groups, whereas a marked reduction of nodules was observed in the TEX‐Liposome‐PTX‐treated group and the combination treatment group (Figure 8a,d). The results again illustrated that the hybridization of TEX endow the excellent lung active targeting ability to liposome, thus enhancing therapy efficacy of PTX against lung metastasis in advanced breast cancer. For recurrent tumors, the tumor volume in all GNR‐containing groups was smaller or even disappeared, exhibiting a significant difference with the control and TEX‐Liposome‐PTX‐treated groups (Figure 8b,d). Excellent antirecurrent tumor efficacy indicated that the recurrent tumors in advanced breast cancer could be treated effectively by in situ thermal ablation triggered by GNR, and a relatively weak antirecurrent tumor efficacy was observed in TEX‐Liposome‐PTX treated group due to the obviously lung targeting capability of TEX.

From the H&E staining of lung slices in control group, metastatic foci, and inflammation could be clearly observed and the alveoli were barely visible with the change of the lung structures (Figure 8d). After treatment with all groups containing TEX‐Liposome‐PTX, the metastatic nodules were significantly reduced, the alveolar structure was restored, and the cotreatment group with GNR and TEX‐Liposome‐PTX showed the best effects. Again, results in H&E staining demonstrated the lung homing affinity of TEX.

Taken together, based on the lung homing ability of TEX and the in situ thermal ablation effect of GNR, the combination strategy of GNR and TEX‐Liposome‐PTX had the expected potential for the treatment of advanced breast cancer.

During the time period of treatment, body weight of mice was recorded every other day to evaluate the safety of the combination strategy. As shown in Figure 8e, body weight in the control group and GNR group showed a slight downward trend. With the rapid progress of tumors, advanced tumors had cachexia, causing the weight loss of mice which without being well treated. After effective treatment, the body weight kept flat, indicating the satisfactory low systemic toxicity in vivo of the combination delivery system.

### 3.8 Study on the immune regulation mechanism of TEX‐Liposome‐PTX combined with PTT

**FIGURE 9:**
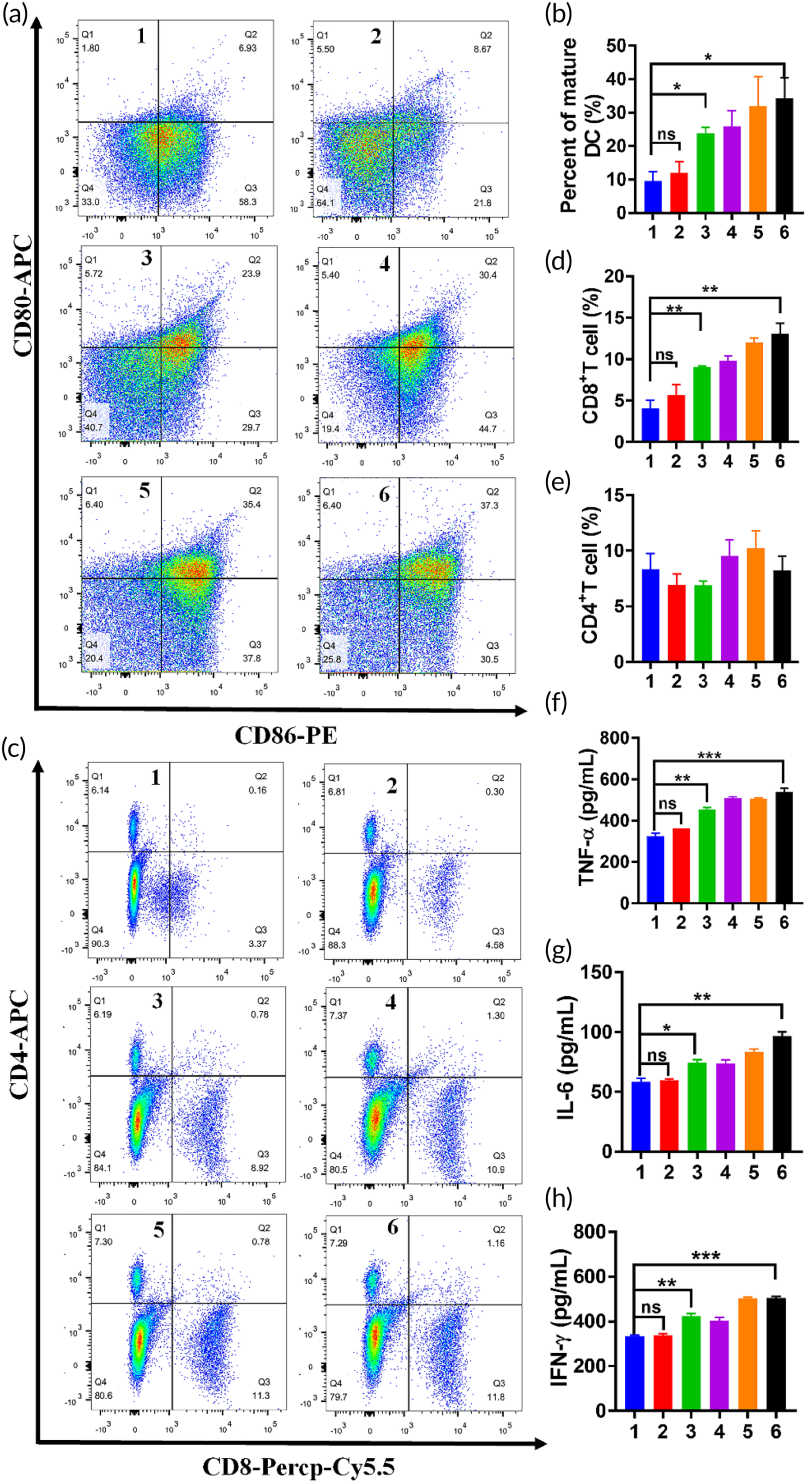
(a) Representative flow cytometry plots of mature DC levels in inguinal lymph nodes treated with saline, TEX‐Liposome‐PTX, GNR, GNR combined PTX, GNR combined Liposome‐PTX, and GNR combined TEX‐Liposome‐PTX. (b) Quantitative analysis of DC maturation level according to (a). (c) Representative flow cytometry plots of lymphocytes differentiation levels in lung after treatment with different formulations. (d) Quantitative analysis of CD8^+^ T lymphocyte cells according to (b). (e) Quantitative analysis of CD4^+^ T lymphocyte cells according to (b). The concentration of cytokines including TNF‐α (f), IL‐6 (g), and IFN‐γ (h) in serum collected from mice (1, control; 2, TEX‐Liposome‐PTX; 3, GNR; 4, GNR + PTX; 5, GNR + Liposome‐PTX; 6, GNR + TEX‐Liposome‐PTX). *p < 0.05, **p < 0.01, ***p < 0.001. DC, dendritic cell; GNR, gold nanorod; IFN‐γ, interferon‐γ; IL‐6, interlekin‐6; PTX, paclitaxel; TEX, tumor‐derived exosome; TNF‐α, tumor necrosis factor‐α

Above results in vivo treatment suggested that the PTT triggered by GNR might have a certain inhibitory effect on lung metastases, which might be caused by spontaneous tumor immunity induced by thermal ablation. According to previous reports, photothermal hyperthermia can trigger the immunogenic cell death to release tumor‐associated antigens, thus promoting the activation of adaptive antitumor immune responses and enhancing the therapeutic efficacy.^20^, ^21^ Thence, the immunoregulatory mechanism of the combination strategy was investigated by detecting the maturation level of DCs in draining lymph nodes, the differentiation of T lymphocytes in lung, and related cytokines in serum. With NIR irradiated to recurrent tumor, GNR‐mediated hyperthermia ablation induced tumor cell apoptosis, which caused the DC recruitment to recurrent tumor for tumor antigen presentation to T cells. Subsequently, CD80 and CD86 expressions in draining lymph nodes were detected to evaluate maturation level of DCs by FCM. As illustrated in Figure 9a,b, there was no significant difference in the maturation level of DC between TEX‐Liposome‐PTX group and control group, while the percent of mature DC in all groups treated with GNR was significantly increased compared with that of control group. These results revealed that chemotherapy could not stimulate DC cells for antigen presentation, while GNR‐mediated thermal ablation exhibited the capability to stimulate the maturation of DCs and generate the immune stimulation capability. For the GNR + TEX‐Liposome‐PTX group, the percent of mature DC was slightly higher than that of GNR group, indicating that the combined therapy promoted the presentation of tumor antigens by DCs and enhanced the tumor‐specific immune effect in vivo.

T lymphocytes are the main force of immunotherapy in vivo.^39^ For immune effect, humoral immunity is mediated by CD4^+^ T cells, and cytotoxic CD8^+^ T lymphocyte can kill tumor cells straightly.^40^ Results of T lymphocyte differentiation in lung tissues were presented in Figure 9c–e. Notably, the proportion of CD4^+^ T cells had no difference in all groups, indicating that GNR and the combination strategy would not activate the humoral immunity mediated by CD4^+^ T cells through MHC‐II. Similarly, the percent of CD8^+^ T cells and the concentration of serum cytokines (TNF‐α, IL‐6, and IFN‐γ) in TEX‐Liposome‐PTX and control groups had no significant difference. While the percent of CD8^+^ T cells in GNR + TEX‐Liposome‐PTX group was twofold higher than that in TEX‐Liposome‐PTX group, suggesting that GNR‐mediated thermal ablation increased the percent of CD8^+^ T cells in lung. In addition, activated CD8^+^ T cells have an increased ability to produce immune cytokines, including IFN‐γ and TNF‐α.^41^ As expected, the cytokine concentration of TNF‐α, IL‐6, and IFN‐γ in GNR + TEX‐Liposome‐PTX group was significantly higher than that in control group, indirectly proving that CD8^+^ T cell‐mediated immunity was activated. In summary, the treatment strategy of GNR combined with TEX‐Liposome‐PTX could activate cell‐specific immune responses through MHC‐I, play a tumor‐killing effect, and enhance the efficacy of local and metastatic tumors.

## 4 CONCLUSION

In summary, we reported a combination strategy composed of biomimetic TEX‐Liposome‐PTX and gold GNR‐PEG for targeted treatment of distant organ metastases and recurrent tumors in advanced breast cancer. In the strategy, GNR‐PEG and TEX‐Liposome‐PTX exhibited excellent anti‐proliferation efficacy against 4T1 cells in vitro, respectively. With the irradiation of NIR, GNR‐PEG cured recurrent tumors through thermal ablation after in situ intratumoral injection, causing the recruitment of DC to present tumor‐derived antigen to CD8^+^ T cells, and subsequently activated the adaptive antitumor immune response. Due to the hybridization of TEX with lung‐PMN targeting capability, the subsequently administrated TEX‐Liposome‐PTX possessed active lung targeting against NIH3T3 cells in vitro and preferentially accumulated in lung due to the specific affinity of homologous exosomes in vivo. Then, PTX was released in lung for targeted chemotherapy of lung metastases tumors with the assistance of adaptive antitumor immune effect mediated stimulated by GNR. In vivo investigation exhibits the enhanced therapy efficacy through the combination of thermal ablation, PTT‐mediated antitumor immune effect, and targeted chemotherapy of PTX for the treatment of advanced recurrent tumors and metastasis breast cancer. Hence, the combination strategy based on hyperthermia therapy based adaptive immunotherapy and TEX‐mediated targeted chemotherapy is a promising therapeutic candidate against advanced breast cancer in clinical.

## CONFLICT OF INTERESTS

The authors declare no conflict of interest.

## AUTHOR CONTRIBUTIONS

Hongyan Zhu, Junxu Li, and Tianqing Liu designed the strategy and performed data analysis. Haiqin Huang, Lanlan Shao, Yan Chen, and Lan Tang carried out the experiments. Haiqin Huang wrote the manuscript. All of the authors have read and approved the final manuscript.

### PEER REVIEW

The peer review history for this article is available at https://publons.com/publon/10.1002/btm2.10284.

## Supporting information


**Appendix** S1: Supporting information.

## Data Availability

The data that support the findings of this study are available from the corresponding author upon reasonable request.
