# Supplementary material for: Synergistic strategy with hyperthermia therapy based immunotherapy and engineered exosomes−liposomes targeted chemotherapy prevents tumor recurrence and metastasis in advanced breast cancer
Source: Bioeng Transl Med. 2021 Dec 27;7(2):e10284. doi: 10.1002/btm2.10284 (PMC9115690; doi:10.1002/btm2.10284)
Supplement: Supplementary file 1 — Appendix S1: Supporting information. [file BTM2-7-e10284-s001.docx]

**Synergistic Strategy with Hyperthermia Therapy Based Immunotherapy and Engineered Exosomes-liposomes Targeted Chemotherapy Prevents Tumor Recurrence and Metastasis In Advanced Breast Cancer**

**Haiqin Huang^a,1^, Lanlan Shao^a,1^, Yan Chen^a^,** **Lan Tang^a^, Tianqing Liu^b,*^, Junxu Li^a,*^, Hongyan Zhu^a,*^**

^a^ *Department of Pharmaceutics, School of Pharmacy, Nantong University, Nantong 226001, China*

^b^ *NICM Health Research Institute, Western Sydney University, Westmead, NSW, 2145, Australia*

* Corresponding authors.

E-mail addresses: [amyntu@126.com](mailto:amyntu@126.com) (H Zhu), junxuli@ntu.edu.cn (J Li), michelle.tianqing.liu@gmail.com (T Liu).

^1^ Equally contributed to this work.

**Table S1.** Hydrodynamic diameter and zeta potential determined by DLS (n=3).

| Samples | Hydrodynamic diameter(nm) | Polydispersity (PDI) | Zeta potential  (mv) |
| --- | --- | --- | --- |
| Liposome | 95.77 ± 0.54 | 0.067 ± 0.009 | -26.69 ± 1.17 |
| Liposome-PTX | 114.42 ± 4.95 | 0.117 ± 0.101 | -34.99 ± 2.22 |
| Exosome | 106.63 ± 1.24 | 0.145 ± 0.028 | -37.45 ± 1.53 |
| Exosome membrane | 336.47 ± 20.33 | 0.220 ± 0.018 | -43.02 ± 2.53 |
| TEX-Liposome (1/100) | 107.85 ± 1.54 | 0.230 ± 0.009 | -28.37 ± 1.13 |
| TEX-Liposome (1/20) | 109.58 ± 1.92 | 0.184 ± 0.024 | -28.64 ± 0.59 |
| TEX-Liposome (1/5) | 121.83 ± 0.96 | 0.172 ± 0.147 | -30.14 ± 2.85 |
| TEX-Liposome-PTX (1/100) | 120.64 ± 1.66 | 0.115 ± 0.048 | -30.30 ± 0.19 |
| TEX-Liposome-PTX (1/20) | 123.82 ± 1.08 | 0.186 ± 0.043 | -35.01 ± 2.41 |
| TEX-Liposome-PTX (1/5) | 133.41 ± 2.50 | 0.228 ± 0.021 | -40.80 ± 1.83 |

**Table S2.** IC_50_ values of PTX, Liposome-PTX and TEX-Liposome-PTX (1/5, 1/20, 1/100) against 4T1 cells

| Groups | IC_50_ (µg/mL) |
| --- | --- |
| PTX | 2.21 ± 0.50 |
| Liposome-PTX | 9.26 ± 0.76 ** |
| TEX-Liposome-PTX (1/5) | 7.87 ± 2.80 ** |
| TEX-Liposome-PTX (1/20) | 10.42 ± 3.15** |
| TEX-Liposome-PTX (1/100) | 10.36 ± 0.97** |

***p*<0.01 vs PTX.


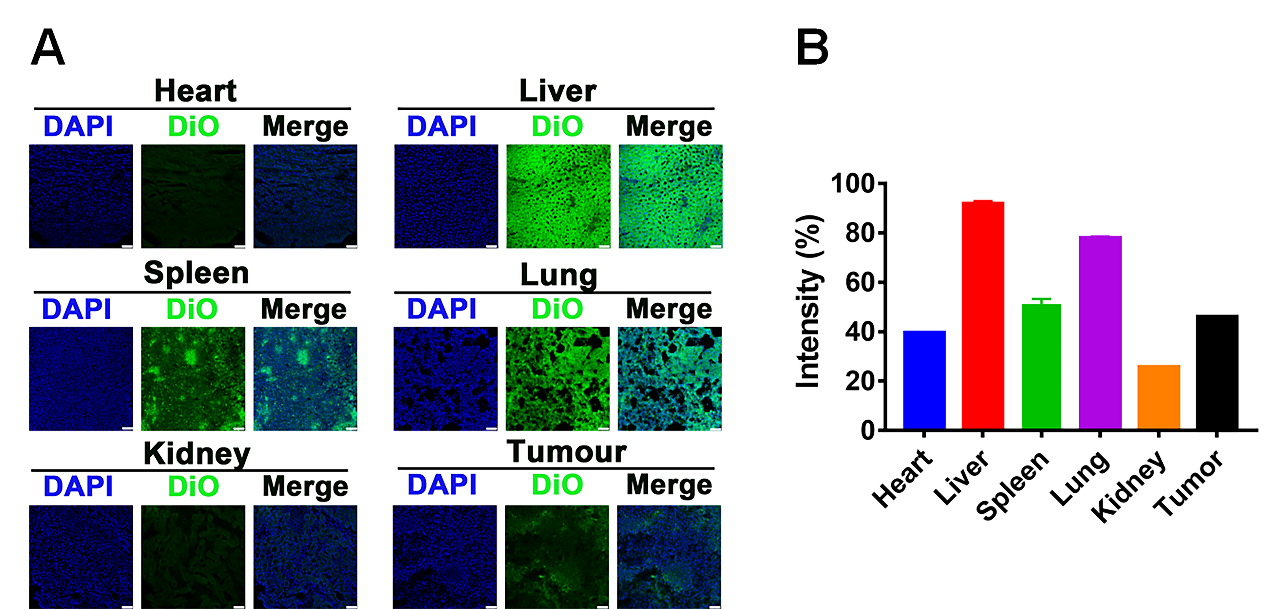


**Figure S1.** (A) CLSM slice images of heart, liver, spleen, kidney, and tumor at the designed time points after treated with TEX-Liposome-PTX (1/20). (B) The semi-quantitative analysis of DiO intensity according to section C. Scale bar of A, 75 µm.


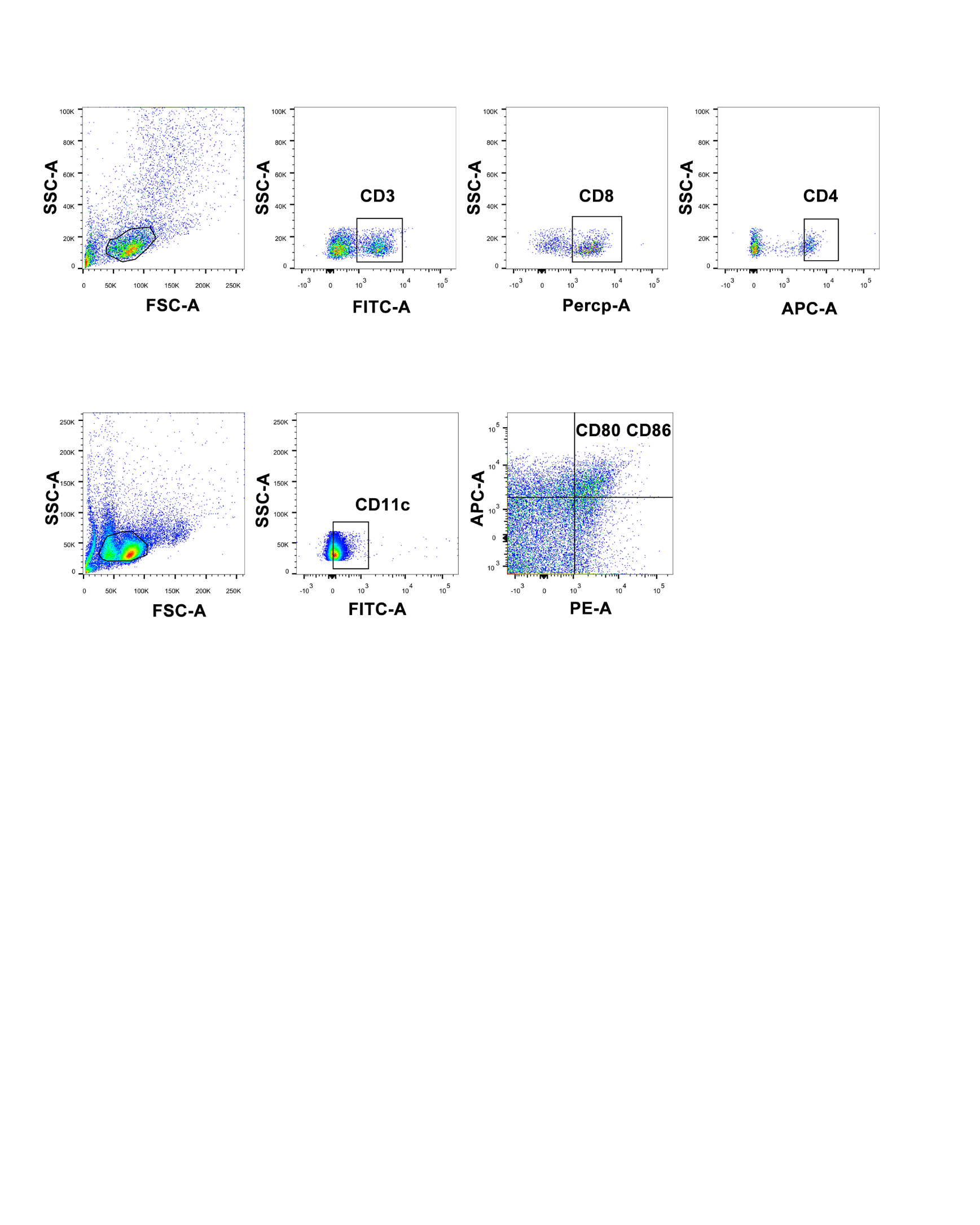


**Figure S2.** Flow cytometry procedure for the identification of mature DC.


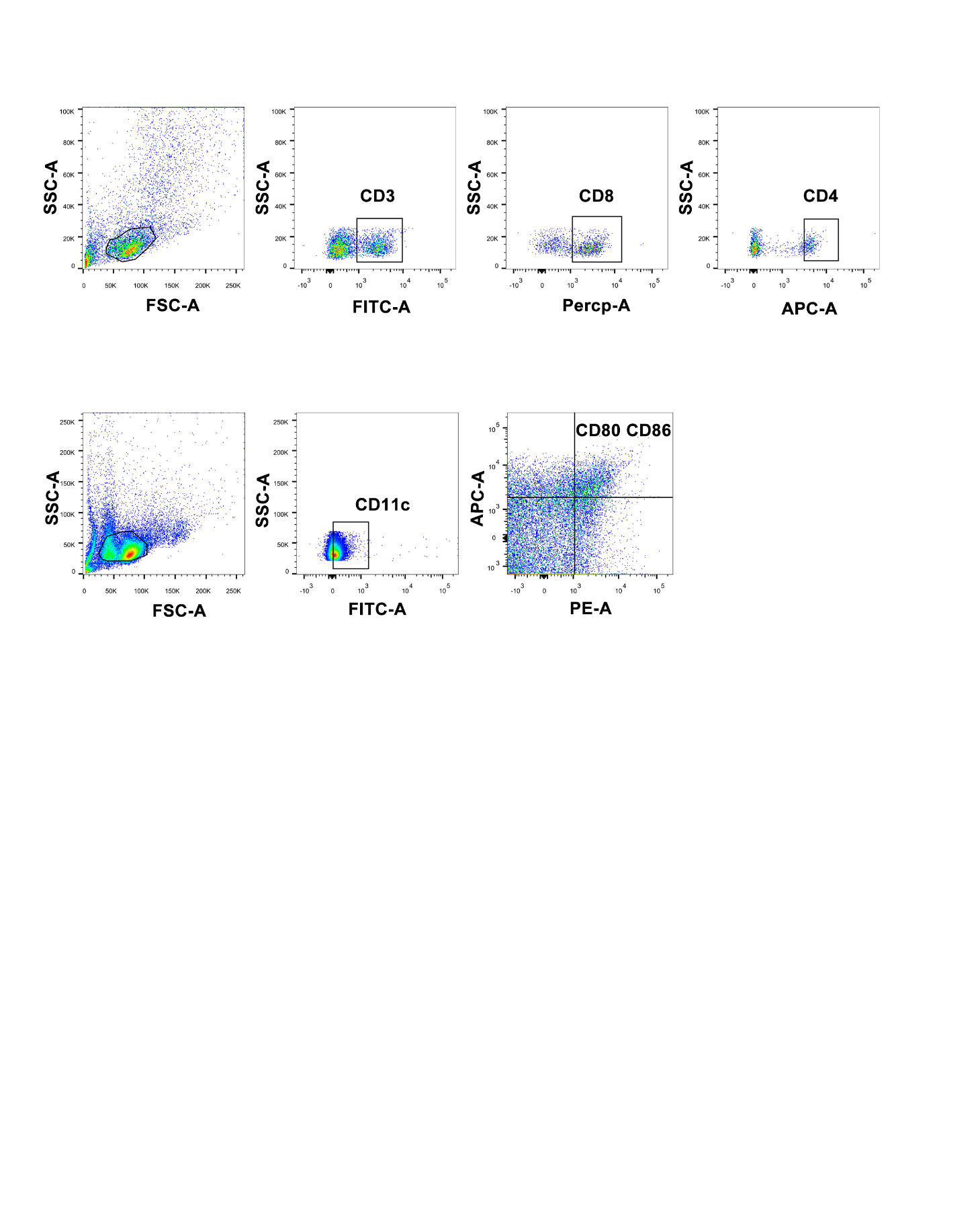


**Figure S3.** Flow cytometry procedure for the identification of lymphocyte.
